# Automated size selection for short cell-free DNA fragments enriches for circulating tumor DNA and improves error correction during next generation sequencing

**DOI:** 10.1371/journal.pone.0197333

**Published:** 2018-07-25

**Authors:** Sabine Hellwig, David A. Nix, Keith M. Gligorich, John M. O’Shea, Alun Thomas, Carrie L. Fuertes, Preetida J. Bhetariya, Gabor T. Marth, Mary P. Bronner, Hunter R. Underhill

**Affiliations:** 1 ARUP Laboratories, Salt Lake City, Utah, United States of America; 2 Huntsman Cancer Institute, University of Utah School of Medicine, Salt Lake City, Utah, United States of America; 3 Department of Pathology, University of Utah, Salt Lake City, Utah, United States of America; 4 Biorepository and Molecular Pathology, Huntsman Cancer Institute, University of Utah, Salt Lake City, Utah, United States of America; 5 Department of Family and Preventive Medicine, Divisions of Genetic Epidemiology and Public Health, University of Utah, Salt Lake City, Utah, United States of America; 6 Department of Human Genetics, University of Utah, Salt Lake City, Utah, United States of America; 7 Department of Pediatrics, Division of Medical Genetics, University of Utah, Salt Lake City, Utah, United States of America; 8 Department of Radiology, University of Utah, Salt Lake City, Utah, United States of America; Broad Institute, UNITED STATES

## Abstract

Circulating tumor-derived cell-free DNA (ctDNA) enables non-invasive diagnosis, monitoring, and treatment susceptibility testing in human cancers. However, accurate detection of variant alleles, particularly during untargeted searches, remains a principal obstacle to widespread application of cell-free DNA in clinical oncology. In this study, isolation of short cell-free DNA fragments is shown to enrich for tumor variants and improve correction of PCR- and sequencing-associated errors. Subfractions of the mononucleosome of circulating cell-free DNA (ccfDNA) were isolated from patients with melanoma, pancreatic ductal adenocarcinoma, and colorectal adenocarcinoma using a high-throughput-capable automated gel-extraction platform. Using a 128-gene (128 kb) custom next-generation sequencing panel, variant alleles were on average 2-fold enriched in the short fraction (median insert size: ~142 bp) compared to the original ccfDNA sample, while 0.7-fold reduced in the fraction corresponding to the principal peak of the mononucleosome (median insert size: ~167 bp). Size-selected short fractions compared to the original ccfDNA yielded significantly larger family sizes (i.e., PCR duplicates) during *in silico* consensus sequence interpretation via unique molecular identifiers. Increments in family size were associated with a progressive reduction of PCR and sequencing errors. Although consensus read depth also decreased at larger family sizes, the variant allele frequency in the short ccfDNA fraction remained consistent, while variant detection in the original ccfDNA was commonly lost at family sizes necessary to minimize errors. These collective findings support the automated extraction of short ccfDNA fragments to enrich for ctDNA while concomitantly reducing false positives through *in silico* error correction.

## Introduction

Circulating cell-free DNA (ccfDNA) testing is an emerging diagnostic approach, allowing for non-invasive, rapid, and real-time testing in oncology and prenatal screening [1]. Although ccfDNA is predominantly of non-neoplastic hematopoietic cell origin [2], solid tissues, including cancers, also contribute to the plasma ccfDNA pool. Such tumor-derived DNA fragments, termed circulating tumor DNA (ctDNA), bear the molecular signatures of the neoplastic cell genome. Relative to microdissection of tumor tissue which interrogates a minute and focal fraction of intratumor genetic diversity, ctDNA is thought to better sample clonal varieties of both primary and metastatic sites through perfusion sampling [3]. However, ctDNA is typically present at very low allele frequencies (median of ~0.5%) due to dilution by abundant normal ccfDNA [4]. As total ctDNA content is correlated with advancing disease stage [5], application of ccfDNA diagnostics for early disease detection will likely require reliable identification of very-low variant allele frequencies (VAFs; i.e., <1%). In addition, intratumoral genetic heterogeneity is common and a key challenge in cancer medicine [6]. Identification of minor subclonal populations is essential for detection of emerging chemoresistance, minimal residual disease, and disease progression.

Size profiling of plasma cell free DNA established the highly fragmented nature of ccfDNA [7]. Fragments are most commonly limited to sizes typical of mononucleosomal and dinucleosomal chromatin degradation products, consistent with an apoptotic origin of ccfDNA [8]. Approaches using paired-end massively parallel sequencing further characterized size-distribution to single-nucleotide resolution, revealing a predominant mononucleosome peak of ~167 bp. In contrast, ctDNA has been observed to consist of shorter fragment sizes compared to ccfDNA. Recently, we demonstrated that isolation of shorter ccfDNA fractions with an offset of 20–50 bp from the mononucleosomal peak of ~167 bp enriched for the *EGFR* T790M variant in three lung cancer patients [9]. While intriguing, this initial study of size selection was confined in scope by sample size, tumor type, and a laborious manual fragment extraction method [9]. Thus, findings did not permit more comprehensive conclusions about the benefits of high-resolution ccfDNA size selection in ctDNA diagnostics. In addition, the study design exclusively used droplet digital PCR (ddPCR) to measure enrichment of known variant alleles. As such, the effects of ccfDNA size selection on variant detection during next generation sequencing (NGS) experiments remains unknown.

NGS enables a broad search for both known and unknown tumor-associated variants including single nucleotide variants, copy number variations, and chromosomal rearrangements [4, 10–12]. However, even the highest fidelity sequencing platforms introduce errors at ≥0.1% [13]. Additional nucleotide changes may be introduced in the PCR amplification steps of sequencing library preparations. Such accumulation of potential false positive errors by sequencing and PCR limits reliable identification of true variants that occur with <1% frequency. Several approaches have been taken to improve very low variant calling by NGS. Ultra-deep sequencing (e.g., >30,000X read depth) improves detection of low frequency variants in ccfDNA [11], but may not be cost-effective for routine diagnostic testing. Alternative or complementary strategies tag individual template molecules with unique molecular identifiers [14, 15], Unique molecular identifiers allow grouping of PCR duplicates arising from the same uniquely tagged native DNA molecule into families. Each family of PCR duplicates produces a single consensus sequence that reduces PCR and sequencing errors through *in silico* error correction. The number of PCR duplicates that yield a single consensus sequence (i.e., family size) is dependent on the number of total reads and the complexity of the library. As such, the reduction of library complexity through size-based isolation of ccfDNA fractions may afford the opportunity to improve error correction through generation of larger family sizes.

In this study, we optimized and implemented a high-throughput-capable automated gel-extraction platform to isolate subfractions of the mononucleosomal peak in sequencing libraries of ccfDNA from patients with melanoma, colorectal adenocarcinoma, and pancreatic ductal adenocarcinoma with confirmed somatic *BRAF* or *KRAS* variants. We sought to determine if selection of shorter ccfDNA fragments increased VAF of tumor-associated variants as detected by ddPCR and NGS. We also studied the NGS data to identify the potential effects of size selection to reduce ccfDNA sample complexity for generating more PCR duplicates (i.e., larger family sizes). We then analyzed the use of incrementally larger family sizes on the occurrence of false positives in the sequencing libraries from healthy controls. Finally, the patient-derived NGS libraries were analyzed to determine whether true VAF remained constant over a wide range of family sizes. In so doing, this study characterizes the potential of automated size-based selection of ccfDNA fractions to improve detection of ctDNA during NGS applications by simultaneously enriching for ctDNA and reducing false positives associated with PCR and sequencing errors.

## Results

### VAFs in ccfDNA determined by ddPCR and NGS are strongly correlated

**Fig 1:**
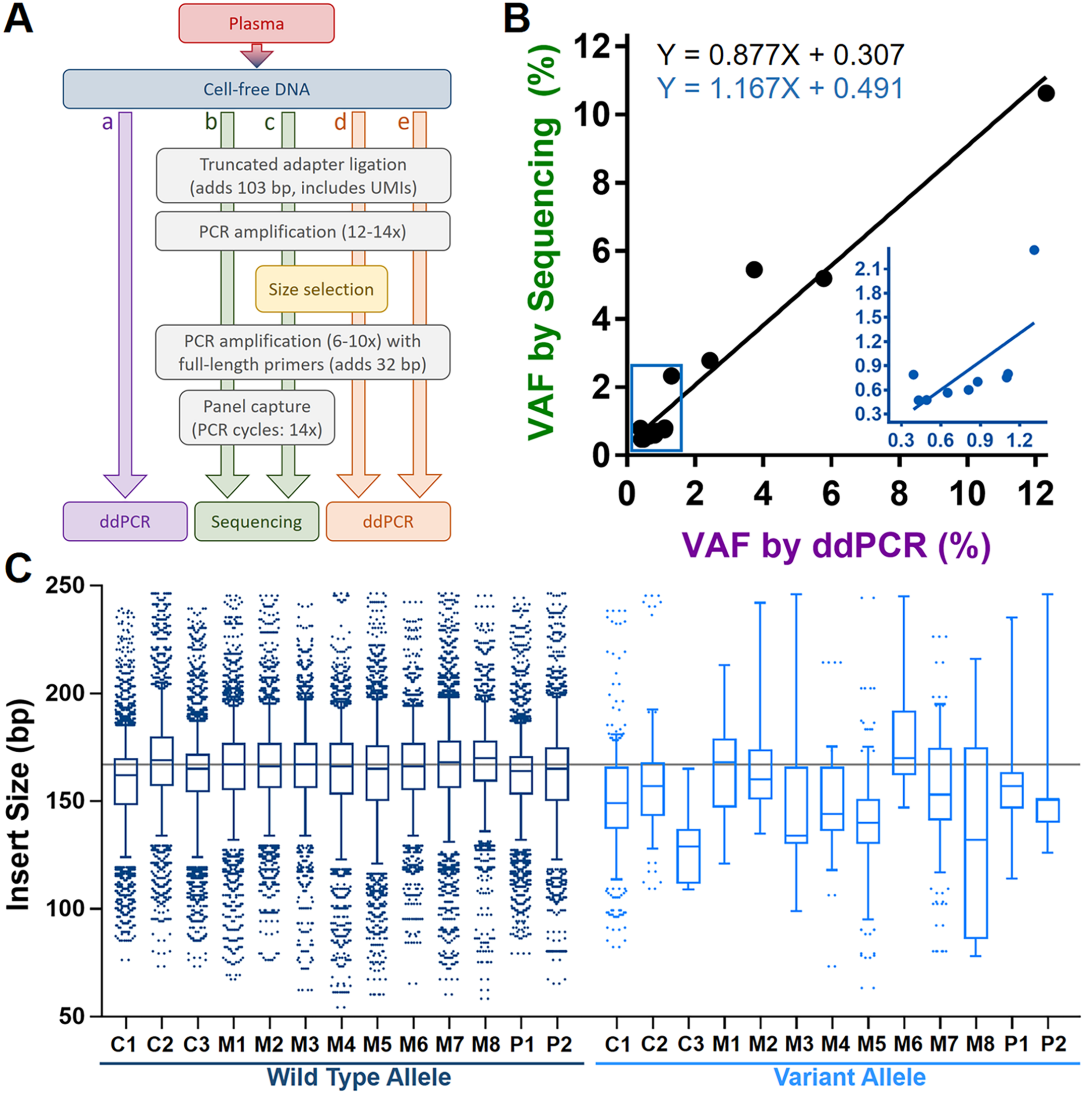
Detection of variant alleles in ccfDNA. Flowchart (A) depicting steps prior to determination of variant allele frequency (VAF). With known variants, VAF can be determined directly from ccfDNA with ddPCR (a), while sequencing requires a multi-step process (b). The addition of truncated adapters followed by extension to full-length in separate steps (b-e) is done to improve resolution during size selection (c, d) of desired subfractions of ccfDNA. There was a strong association (B) between direct measurement of VAF in ccfDNA by ddPCR (A-a) and by the multi-step sequencing process (A-b). This association was present even at VAFs < 1.5% (B-inset). The equations for each colored regression line are shown in a corresponding color. In (C), boxplots of wild type alleles (dark blue) and variant alleles (light blue) by NGS are shown for each cancer patient (C = colorectal adenocarcinoma; M = melanoma; P = pancreatic ductal adenocarcinoma). In (C), data are only shown for insert sizes ≤250 to focus results on the mononucleosome as that length approximates the midpoint between the mononucleosome and dinucleosome lengths associated with ccfDNA. The light gray line identifies the median insert size (167 bp) from all patients. In the majority of patients, the median insert size of the tumor-associated variant allele was shorter than the corresponding wild type allele.

High-resolution size selection of ccfDNA libraries prior to sequencing involves multiple PCR amplification steps (Fig 1A). Therefore, we first sought to determine whether the library preparation process or subsequent PCR amplification steps result in drift of the detectable VAF. Samples from 13 patients with a *BRAF* or *KRAS* variant present in solid tumor tissue from melanoma (*N* = 8), colorectal adenocarcinoma (*N* = 3), or pancreatic ductal adenocarcinoma (*N* = 2) and a corresponding quantifiable variant present in ccfDNA by ddPCR were analyzed (Table 1). Droplet digital PCR performed on ccfDNA prior to ligation of adapters and library formation determined VAF (Fig 1A) and facilitated direct molecular counting of amplifiable unique wild type (WT) and variant counts (S1 Table, S1 Fig). After addition of truncated adapters with unique molecular identifiers, subsequent extension to full-length adapters, panel capture, and multiple PCR amplification steps, WT counts, variant counts, and VAF were determined by NGS (Fig 1A). Each NGS count (either WT or variant) for all reported results hereafter is an aligned consensus read derived from PCR duplicates with the same unique molecular identifier. Thus, each count represents a single unique molecule from the original ccfDNA sample prior to library PCR amplification. To evaluate for losses associated with NGS, WT and variant counts obtained from ddPCR were extrapolated to determine the expected number of WT and variant counts from NGS assuming a lossless system for a given amount of ccfDNA library input (S1 Table). There was a significant correlation between the extrapolated ddPCR counts and NGS counts for WT alleles (Pearson’s *r* = 0.72, *P* = 0.005) and variant alleles (*r* = 0.96, *P* < 0.001). For both WT and variant counts an increased number of counts was detected by NGS over ddPCR for most of the samples. That difference was statistically significant for the WT alleles (57.5±86.7%, *P* = 0.034; S2A Fig), but not variant alleles (77.1±162.9%, *P* = 0.11; S2B Fig). Undercalling of ddPCR is likely a reflection of the requirement of detectable DNA fragments to contain intact amplicon regions. Such non-amplifiable alleles may be detectable by NGS after hybrid capture. Conversely, loss of allele counts detectable by NGS could be a consequence of inefficient adapter ligation, post ligation cleanup, or non-uniform hybrid capture. While both methods may be subject to losses and regardless of absolute count differences, the VAF was strongly correlated between ddPCR and NGS (*r* = 0.97, *P* < 0.001; Fig 1B). In the subset of samples with VAF <1.5% by ddPCR the association persisted (*r* = 0.68, *P* = 0.046; Fig 1B, inset). Thus, sample preparation and analysis by NGS used in this study did not adversely affect VAF in ccfDNA as corroborated by ddPCR.

**Table 1:** Demographics and variant allele frequency (VAF).

ID	Cancer Type	Age, yrs	Gender	Stage	Allele	VAF by ddPCR, %
C1	Colorectal	69	M	IV	*KRAS* G13D	12.26
C2	Colorectal	78	F	III	*BRAF* V600E	2.43
C3	Colorectal	63	M	IV	*KRAS* G12D	0.65
M1	Melanoma	49	F	IIIC	*BRAF* V600E	0.81
M2	Melanoma	80	M	IIIC	*BRAF* V600K	1.11
M3	Melanoma	53	F	IV	*BRAF* V600E	0.39
M4	Melanoma	45	M	III	*BRAF* V600E	1.31
M5	Melanoma	67	F	IV	*BRAF* V600E	3.74
M6	Melanoma	67	M	IV	*BRAF* V600K	0.88
M7	Melanoma	39	M	IV	*BRAF* V600E	5.78
M8	Melanoma	50	M	IV	*BRAF* V600K	1.10
P1	Pancreatic	63	M	IV	*KRAS* G12D	0.48
P2	Pancreatic	54	F	IV	*KRAS* G12V	0.43

### Automated size selection reproducibly isolated ccfDNA subfractions

**Fig 2:**
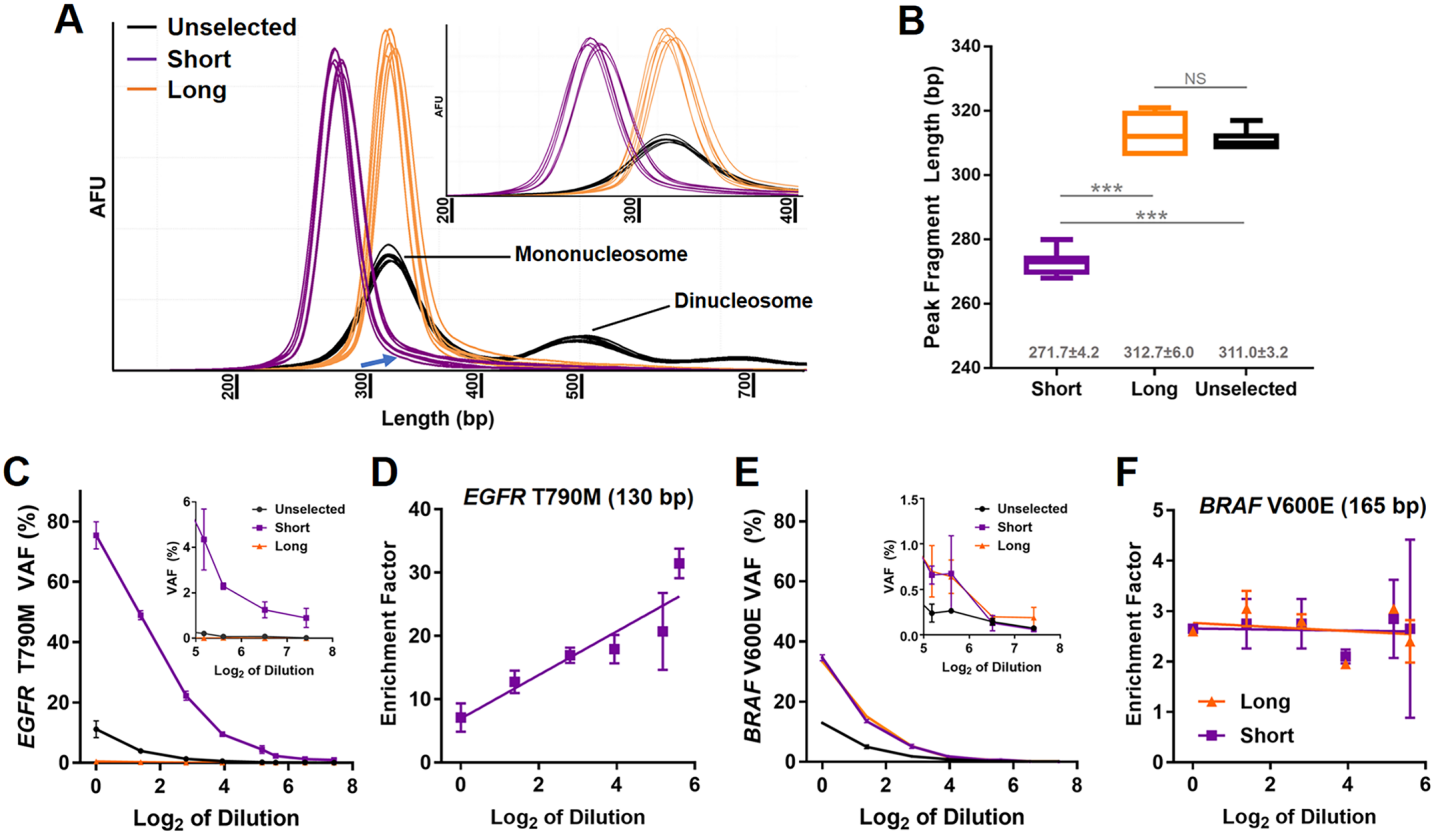
Effect of size selection on VAF in spiked ccfDNA libraries using an automated gel-extraction platform. Distribution by densitometry of the short (purple) and long (orange) fractions isolated from healthy control unselected ccfDNA samples (black; *N* = 7) is shown in (A). Size includes full-length adapters (~135 bp). Note the evidence of a tail in the short fraction (A, blue arrow) consistent with longer fragments migrating with a shorter target fragment size. Although the overall distributions overlapped, the peak fragment length of the short fraction was significantly less than the long fraction (B). No significant difference was measured between the peak fragment lengths of the long fraction and the unselected mononucleosome (B). Gray numbers indicate the mean±SD peak fragment length for each sample (B). VAF determined by ddPCR for the *EGFR* T790M synthetic spike (130 bp) for the short (purple) and long (orange) fractions and the unselected ccfDNA (black) are graphed in (C). In the short fraction the T790M allele remained detectable even when it was undetectable in unselected ccfDNA (C, inset), while it was virtually absent from the long fraction regardless of dilution. The enrichment factor in the ‘small’ fraction was associated with extent of dilution with the greatest amount of enrichment occurring in the most diluted samples (D; data only shown when unselected ccfDNA VAF was above the limit of blank by ddPCR). VAF by ddPCR for the *BRAF* V600E synthetic spike (165 bp) is shown in (E). Overall, there was a general trend towards enrichment in both the short (E, purple) and long (E, orange) fractions. The variant was present throughout the short samples except at the lowest dilutions (E, inset). Extent of enrichment was relatively consistent regardless of dilution (F). In A-D, error bars indicate standard deviation from independent duplicate experiments. *** *P* < 0.001; NS = not significant; AFU = arbitrary fluorescent unit.

We have previously shown in *EGFR*-mutant lung cancer samples that VAF increased in association with shorter ccfDNA fragments [9]. Here, we observed in 13 patients that the median insert sizes corresponding to the mononucleosome (< 250 bp) associated with the variant alleles present in either *BRAF* or *KRAS* were generally shorter than the corresponding wild type alleles (151.8±12.8 vs. 166.9±2.5 bp, respectively; *P* = 0.001; Fig 1C). Individual beeswarm plots for each patient for both WT and variant are shown in S3 Fig (insert size < 250 bp, mononucleosome) and S4 Fig (insert size > 250 bp, dinucleosome and larger). With the goal of enriching for variant alleles, we then optimized and tested an automated agarose gel-based extraction method for the consistent selection of size-based ccfDNA fractions (Fig 2A and 2B). Subsequently, enrichment capabilities of gel-based ccfDNA fragment size selection were established using pooled ccfDNA from healthy controls spiked with synthetic *EGFR* T790M fragments (length 130 bp) and *BRAF* V600E fragments (length 165 bp) at similar VAF. Truncated adapters were added, PCR amplified to produce libraries of spiked and unspiked ccfDNA, and then mixed to yield an eight sample dilution series of unselected spiked ccfDNA libraries with VAFs ranging from 0.01% to 13.1% as measured by ddPCR (S5 Fig). Short and long fractions were then extracted from 1 μg of PCR-amplified truncated ccfDNA libraries to target the *EGFR* and *BRAF* spike-in variants, respectively. Full-length libraries were generated from size-selected fractions and VAF was determined by ddPCR (S5 Fig). Isolation of the short fraction increased VAF of the *EGFR* T790M variant (130 bp) in each dilution (Fig 2C). There was a strong association between dilution factor and enrichment (Pearson’s *r* = 0.92, *P* = 0.009; Fig 2D) indicating greatest enrichment occurred at the lowest VAF. The *EGFR* T790M variant was absent in the long fraction (Fig 2C). The VAF of the *BRAF* V600E variant (165 bp) was consistently greater than the unselected VAF in both the short and long fractions (Fig 2E), but the extent of enrichment remained relatively constant across all dilutions (Fig 2F). As such, the enrichment observed for the 165-bp *BRAF* V600E variant was likely due to elimination of wildtype *BRAF* found in the dinucleosomal and larger plasma DNA components. Collectively, these findings characterize electrophoretic mobility of ccfDNA under the prescribed experimental conditions. Specifically, for a given size of ccfDNA the distribution is not fully Gaussian. For a targeted size range, longer rather than shorter fragments outside the desired range are more likely to be present as exemplified by the absence of the *EGFR* T790M variant (130 bp) in the long fraction and the presence of the *BRAF* V600E variant (165 bp) in the short fraction. This is further shown in the densitometry plots where a tail of longer fragment sizes is present in the short fraction (Fig 2A, arrow). These results also support the automated agarose gel-based extraction method for reproducibly and accurately separating subpopulations of the mononucleosomal ccfDNA component after NGS library preparation.

### Automated selection of shorter ccfDNA fragments increased VAF

**Fig 3:**
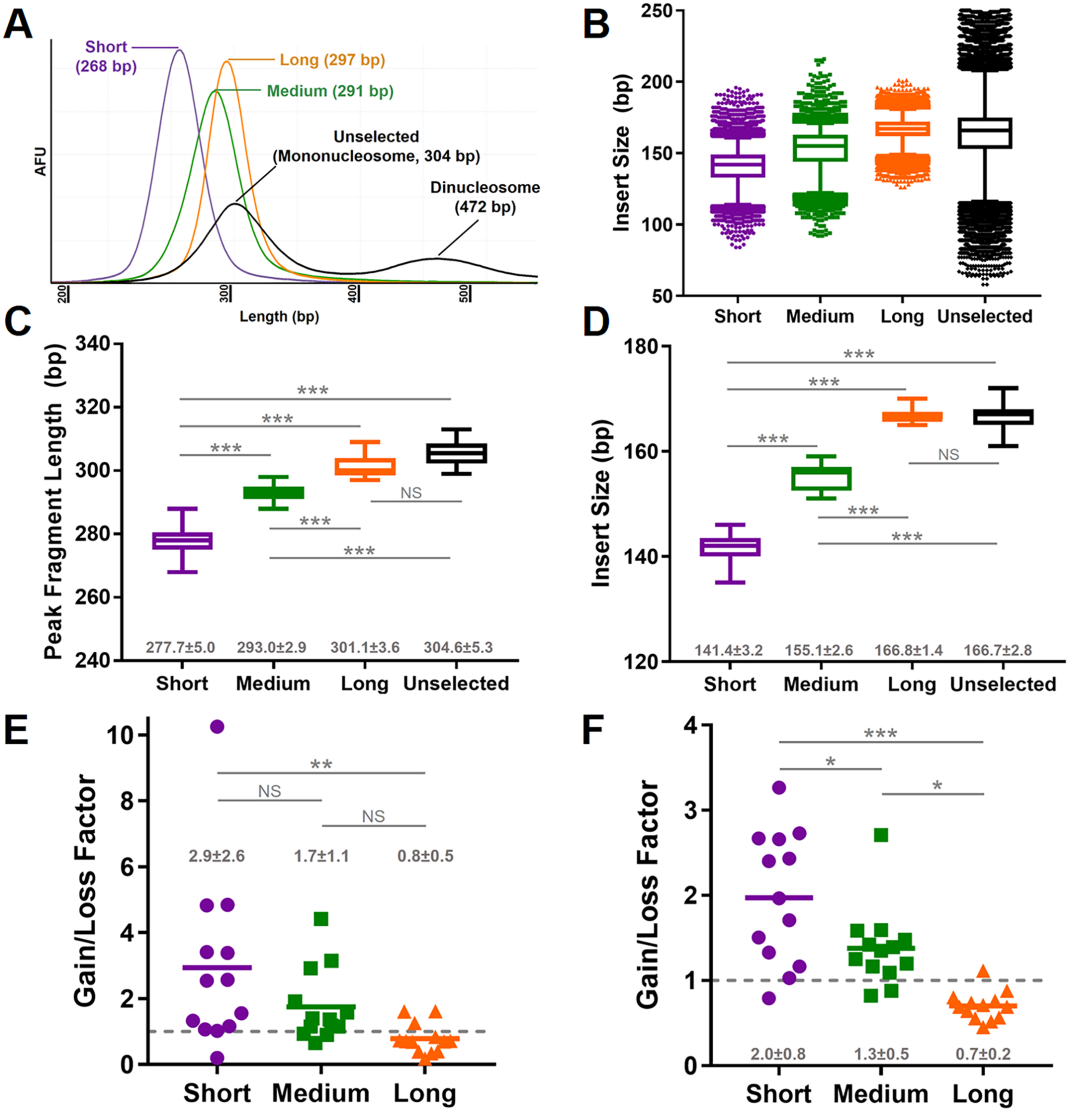
Enrichment of variant alleles in short ccfDNA fractions. In (A), representative distributions by densitometry are shown of the isolated fractions (short—purple; medium—green; long—orange) from the original ccfDNA (black) of a single cancer patient. The fragment lengths include full-length adapters (~135 bp). The cumulative distribution of insert sizes at variant locations from all patients for each subfraction (B) show a profile consistent with densitometry (A). The peak fragment lengths from each patient by densitometry (C) and the median insert size by sequencing (D) were statistically significantly different between each respective subfraction, while observations for the long fraction were similar to the unselected mononucleosome (black). Enrichment for variant alleles was greatest in the short fraction by both ddPCR (E) and sequencing (F) with intermediate enrichment in the medium fraction (F). In the long fraction analyzed by both modalities there was a tendency for reduction in VAF (E and F). Solid bars represent the mean value. In (C-E), mean±SD values are shown in gray. * *P* < 0.05; ** *P* ≤ 0.01; *** *P* ≤ 0.001; NS = not significant; AFU = arbitrary fluorescent unit.

We then isolated size-based fractions from the ccfDNA of patients with solid tumors (Table 1) to characterize the effects on VAF as quantified by both ddPCR and NGS. Both short and long fractions were extracted from PCR-amplified truncated ccfDNA libraries (1 μg; Fig 3A). In a second independent run of PCR-amplified truncated ccfDNA libraries (1 μg), an intermediate fraction (i.e., medium) targeted between the short and long fractions was also isolated (Fig 3A). The distribution of sequencing-derived insert sizes from all short, medium, and long fractions, and unselected libraries are shown in Fig 3B, which demonstrates a substantial amount of overlap between fractions. However, there was a statistically significant difference between fractions for both the peak fragment length by densitometry (*F*(3,48) = 99.4, *P* < 0.001; Fig 3C) and the median insert size by NGS (*F*(3,48) = 283.9, *P* < 0.001; Fig 3D) indicating distinct subfractions of ccfDNA were isolated from the original mononucleosome distribution of fragment sizes. There was a significant difference in the change of VAF between sub-fractions relative to the unselected library as determined by ddPCR (*F*(2,36) = 5.4, *P* = 0.009; Fig 3E and S6 Fig) and sequencing (*F*(2,36) = 17.7, *P* < 0.001; Fig 3F). A significantly larger increase was present in VAF for the short fractions compared to the long fractions by both ddPCR (2.9±2.6 vs. 0.8±0.5 fold-change, respectively; *P* = 0.006) and NGS (2.0±0.8 vs. 0.7±0.2 fold-change, respectively; *P* < 0.001). In the NGS data, there was also an increase in VAF from the short fractions compared to the medium fractions (2.0±0.8 vs. 1.3±0.5 fold-change, respectively; *P* = 0.015) and the medium fractions compared to the long fractions (1.3±0.5 vs. 0.7±0.2 fold-change, respectively; *P* = 0.013). Thus, selection of shorter ccfDNA fragments increased VAF and exclusion of longer ccfDNA fragments did not adversely affect VAF.

The potential source of enrichment was explored using NGS data as each WT or variant allele count was derived from a consensus read representing a unique ccfDNA molecule. WT and variant counts are shown in S2 Table for the unselected ccfDNA and the short, medium, and long ccfDNA fractions. Using the data in S2 Table, the percent difference for each fraction relative to the unselected ccfDNA WT and variant counts was determined to identify effects of size selection on gain/loss of counts. Between ccfDNA subfractions there was a significant difference in WT counts (*F*(2,36) = 42.6, *P* < 0.001; S7A Fig). The short ccfDNA fraction demonstrated the greatest reduction of WT counts at a mean of 48.2±17.1%. In contrast, WT counts in the long ccfDNA fraction was relatively unchanged at an increase of 1.3±9.1%. For variant counts, there was also a significant difference (*F*(2,36) = 6.9, *P* = 0.003; S7B Fig) between subfractions largely due to the 28.6±21.4% reduction in variant counts present in the long ccfDNA fraction. The variant counts in the short ccfDNA fraction was relatively unchanged at an increase of 0.3±37.3% indicating loss of few variants during size selection. Of note, we also observed within each subfraction of ccfDNA a tendency for the variant alleles to have shorter insert sizes and a broader distribution compared to WT alleles (S8 Fig). These findings in combination with earlier observations from unselected ccfDNA (Fig 1C) support a greater proportion of ctDNA at shorter ccfDNA fragment lengths. Thus, isolation of short ccfDNA fragments enriched for ctDNA through reduction of WT alleles without compromising variant allele detection.

### Automated size selection of ccfDNA fragments generated larger family sizes

**Fig 4:**
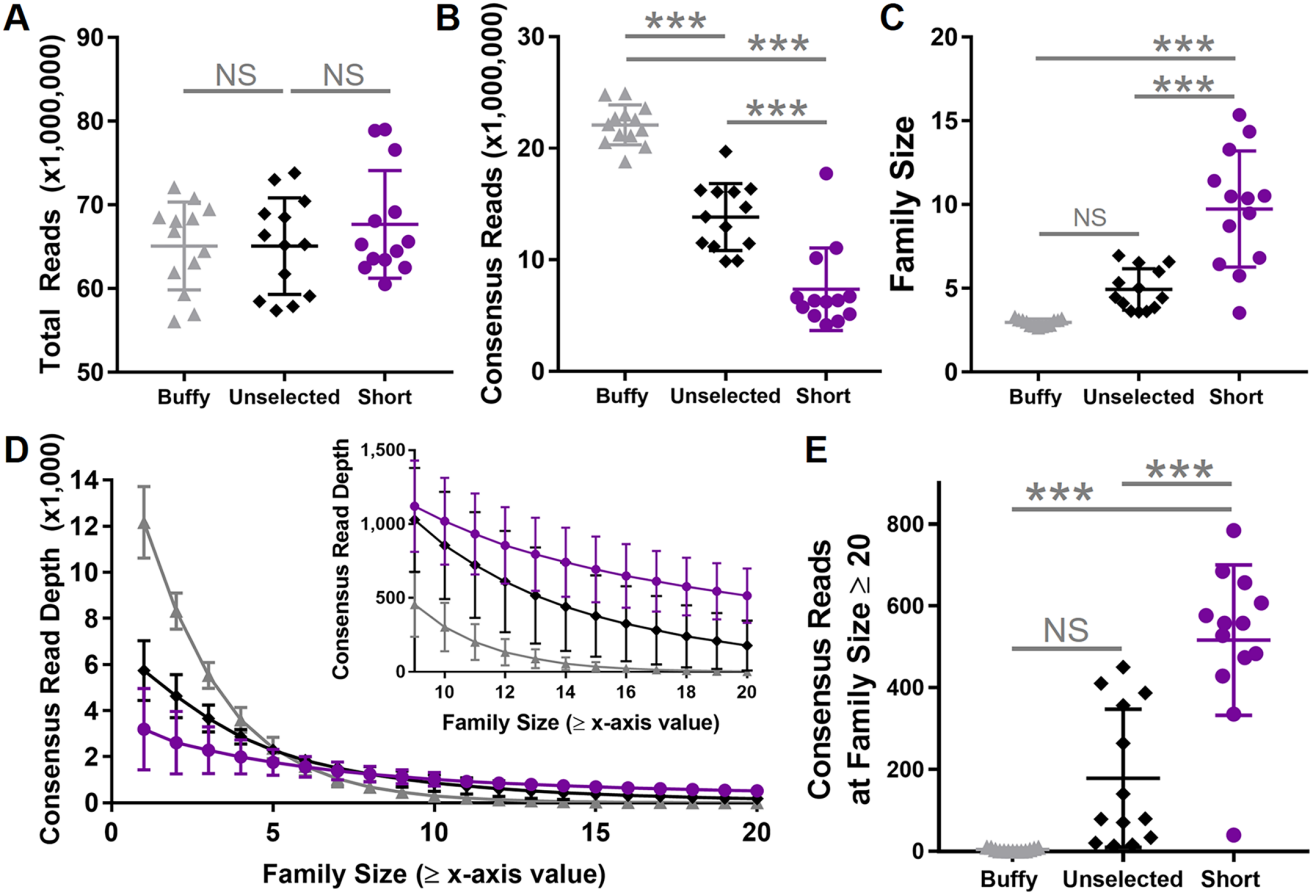
Generation of large family sizes in short ccfDNA. Total reads were similar between sheared buffy coat DNA, unselected ccfDNA, and short ccfDNA (A). Consensus read depth (family size ≥1) was greatest in buffy coat DNA, followed by unselected ccfDNA, and then short ccfDNA (B). Average family size was greatest in the short ccfDNA (C). At the specific variant locations for each patient, consensus read depth in buffy coat DNA rapidly decayed, reaching zero by family size ≥20 (D, gray). In contrast, both the unselected ccfDNA (D, black) and the short ccfDNA (D, purple) showed fewer consensus reads at family size ≥1, but maintained a greater read depth at larger family sizes (D, inset). Consensus read depth at family size ≥20 was greatest in short ccfDNA (E). In (A-C) and (E), solid bars represent the mean value. In (A-E), whiskers correspond to the standard deviation. *** *P* ≤ 0.001; NS = not significant.

We next considered the effects of *a priori* physical size selection on read depth and family size. For all locations there was a statistically significant difference in total reads amongst all sample types (*F*(4,60) = 6.4, *P* < 0.001), which was solely attributable to a modest increase in the long ccfDNA fraction (Fig 4A, S9A Fig). This is an important starting point as the similarity in total reads between different samples indicates the subsequent findings are not due to experimental bias. A statistically significant difference in consensus aligned reads between groups was identified (*F*(4,60) = 38.0, *P* < 0.001). Buffy-coat DNA had the greatest number of consensus aligned reads, while short ccfDNA had the fewest (Fig 4B, S9B Fig). The on-target fraction was significantly different between groups (*F*(4,60) = 6.4, *P* < 0.001) largely due to a slight lowering of the on-target fraction in short ccfDNA (S9C Fig). Average family size was also significantly different between sample types (*F*(4,60) = 20.1, *P* < 0.001). Average family size was largest in the short ccfDNA fraction and smallest in buffy coat DNA (Fig 4C). The family sizes for medium and long ccfDNA fractions were significantly larger than buffy coat DNA (S9D Fig). As such, the reduction in sample complexity through isolation of ccfDNA mononucleosome fractions yielded larger average family sizes for a similar number of total reads even in the context of a reduced on-target fraction in the short ccfDNA fraction. This effect was then evaluated at the known variant locations. Although buffy coat DNA had the largest consensus read depth at family size ≥1, there was a rapid reduction with increasingly larger family sizes (Fig 4D). Consensus read depth decayed more slowly for unselected ccfDNA and short ccfDNA (Fig 4D). At family size ≥20, there was a statistically significant difference in consensus read depth between sample types at the variant locations (*F*(4,60) = 24.8, *P* < 0.001; S9E Fig). The short, medium, and long ccfDNA fractions demonstrated the greatest consensus read depth at family size ≥20 (Fig 4E; S9E Fig).

### Larger family sizes reduced false positives

**Fig 5:**
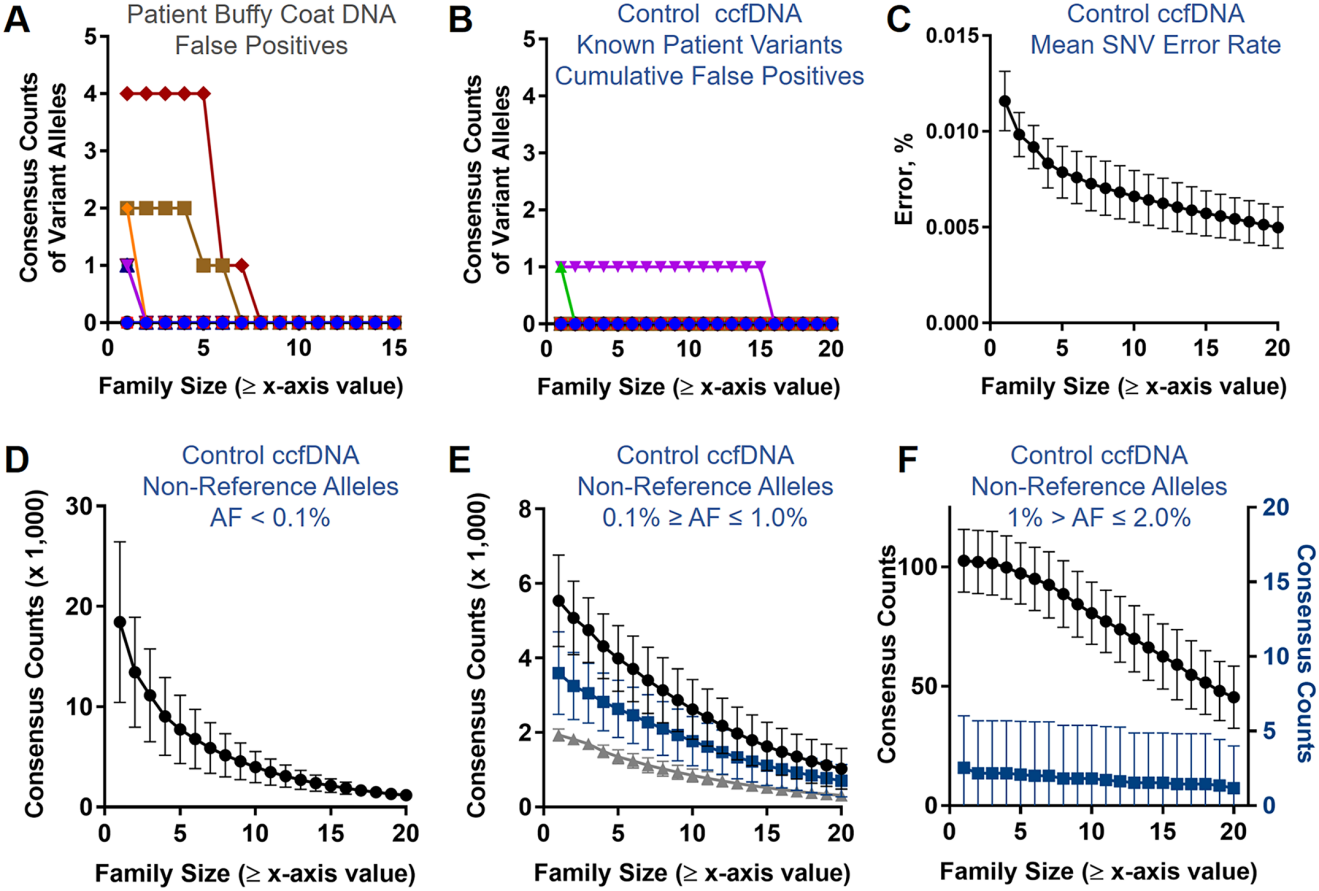
Reduction of false positives at larger family sizes. Corresponding variants present in patient ccfDNA were queried in matched buffy coat DNA (A). False positives were few and incrementally decreased with larger family sizes (A). In (B), the cumulative number of false positives from all healthy control ccfDNA and all five targeted patient variants is shown. Overall, only two false positives were identified. In (C), the mean error rate across the entire capture panel (128 genes, 128 kb) decreased with increasingly larger family sizes. Total consensus aligned counts for non-reference alleles with AF < 0.1% (D), 0.1% ≥ AF ≤ 1.0% (E), and 1.0% > AF ≤ 2.0% (F) are shown (black circles). In (E) and (F), non-reference alleles are sub-categorized as “unique” (blue squares) or “shared” (gray triangles). In (F), “shared” non-reference alleles are not shown as they are similar to the total count. In (F), the “unique” non-reference allele count is plotted on a second y-axis. In (C-F), whiskers correspond to the standard deviation.

We then examined the association between family size and false positives. During a targeted search for the corresponding known variant in the buffy coat DNA from each patient, few false positives were identified (Fig 5A). We then analyzed unselected ccfDNA from 11 healthy controls sequenced under identical conditions. As with the patient samples, the greater of 10 ng or 1 mL plasma equivalent of ccfDNA was used for the initial library input. The mean amount of ccfDNA present in the healthy controls was 11.6±4.7 ng/mL plasma (median: 11.3 ng/mL plasma; range: 3.8–15.3 ng/mL plasma). While there was a trend for a larger amount of ccfDNA per mL plasma in patients, the difference was not statistically significantly greater than the controls most likely due to the large variation in the patient data and sample size associated with each cohort (20.1±14.5 vs. 11.6±4.7 ng/mL plasma, respectively; *P* = 0.07). We similarly found few false positives for the known patient variants in healthy control ccfDNA (Fig 5B). In both patient buffy coat DNA and control ccfDNA the allele frequency for known variants was < 0.01% suggesting that constrained searches of known variants may be associated with a low error rate. Of note, we also observed that family size in the control unselected ccfDNA was significantly larger than patient unselected ccfDNA (8.4±2.7 vs. 4.9±1.2, respectively; *P* < 0.001), which could not be explained by differences in total reads or on-target fractions (S10 Fig). This latter finding supports the supposition that reduction in sample complexity through size selection generates larger family sizes as patient-derived ccfDNA is expected to be more complex than control ccfDNA due to contributions from tumor cells, higher concentration of ccfDNA present in plasma, or both.

Next, we evaluated the aligned base error rate in the control ccfDNA to study occurrence of false positives during untargeted searches using the 128 gene (128 kb) panel (S3 Table). Globally, at family size ≥ 1 the mean error rate was 0.011±0.002% and there was a reduction in error with incrementally larger family sizes (Fig 5C). At family size ≥ 20 the mean error rate was significantly reduced by 57.0±7.7% (*P* < 0.001). Although the global mean error rate provides an overall metric for quality of sequencing, the principal source of false positives during NGS detection of very low frequency variants are due to local errors associated with stochastic noise or position-specific common errors. Locally, non-reference allele counts in control ccfDNA similarly reduced with incrementally larger family sizes (Fig 5D–5F). At a non-reference allele frequency ≥ 0.1% the data was parsed into “unique” and “shared” locations. A shared location was defined as the presence of a non-reference allele in at least three control ccfDNA samples, thus unique locations were representative of stochastic noise. The majority of non-reference alleles detected at a frequency ≥ 0.1% and ≤ 1.0% were due to unique rather than shared locations (Fig 5E). Within this frequency range there was a significant reduction of 81.6±7.3% (*P* < 0.001) in unique non-reference allele counts between family size ≥ 1 and family size ≥ 20. Non-reference alleles detected at a frequency > 1% and ≤ 2.0% were relatively few and largely due to shared locations (Fig 5F). However, it is notable that on average there were ~2 non-reference unique variants in the control ccfDNA with a frequency > 1% and ≤ 2.0% present even at large family sizes (Fig 5F). Combined, these findings indicate stochastic sequencing noise and/or PCR errors may confound identification of true variant alleles during untargeted searches. Regardless, the control data provides compelling evidence that generation of large family sizes improves *in silico* error reduction.

### VAF remained constant in shorter ccfDNA fractions at larger family sizes

**Fig 6:**
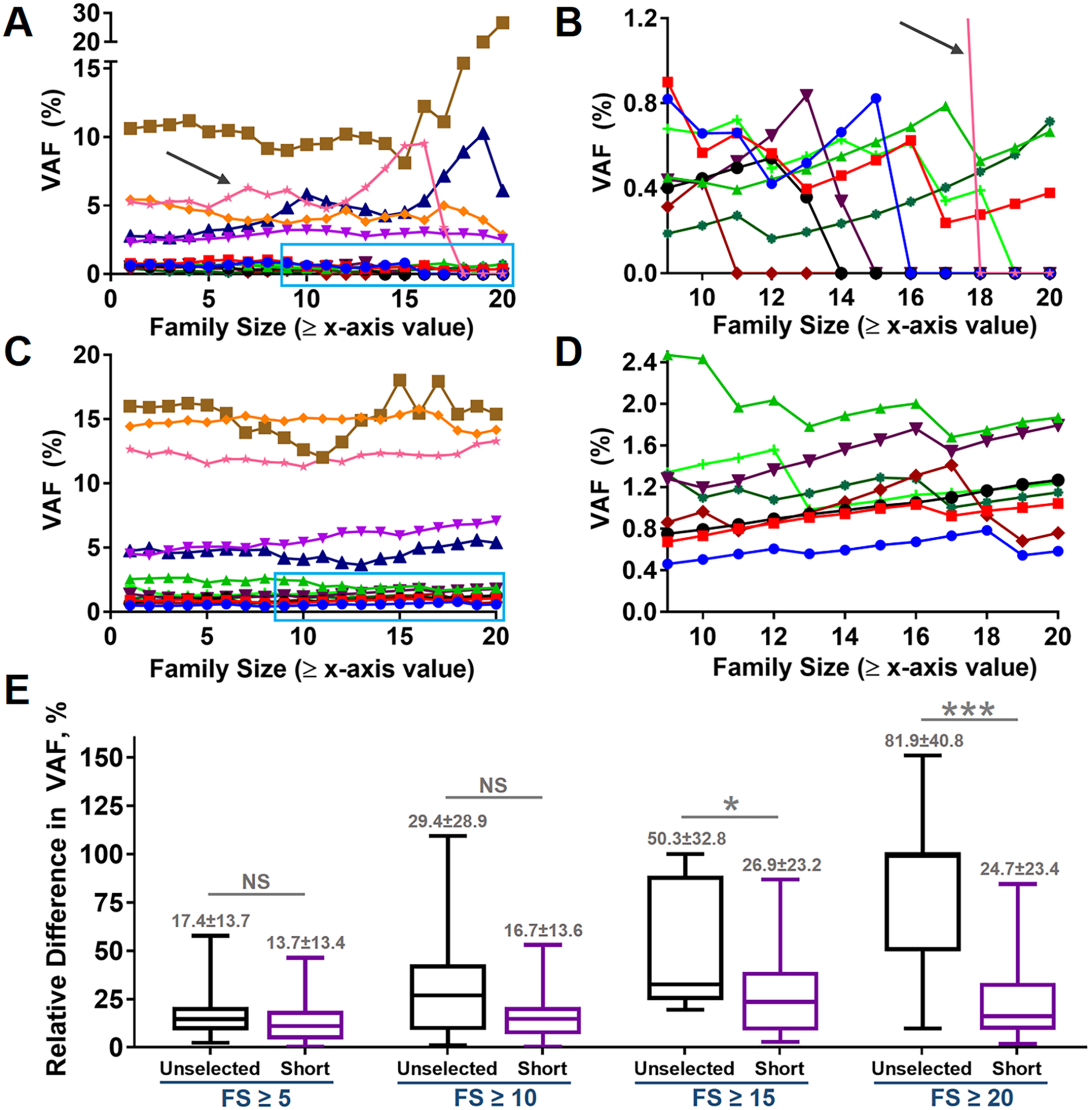
Effects of family size on VAF. Overall, VAF was relatively stable up to a family size ≥10 in unselected ccfDNA (A). However, at larger family sizes VAF became less stable and included complete loss of variants in some samples (B, magnification of area in blue box shown in A). Of note, complete loss of the variant allele occurred in one sample with an initial VAF > 5% (A and B, black arrow). In contrast, VAF remained relatively stable up to family size ≥20 in the short ccfDNA fraction (C, magnification of area in blue box shown in D). Note the apparent increase of VAF in the short ccfDNA fraction at lower allele frequencies (D) compared to the unselected ccfDNA (B). The relative percent difference in VAF was similar in unselected and short ccfDNA at family size (FS) ≥5 and FS ≥10 (E). However, the relative percent difference was statistically significantly lower in the short ccfDNA fraction at FS ≥15 and FS ≥20 (E). * *P* < 0.05; *** *P* ≤ 0.001; NS = not significant.

Finally, we studied the effects of family size on VAF since larger family sizes were associated with a reduced consensus read depth. In the unselected ccfDNA, VAF remained relatively constant up to family size ≥10; however, VAF subsequently became inconsistent at larger family sizes (Fig 6A and 6B). In ~46% of patients (6 of 13) the variant allele was lost before family size ≥20 (Fig 6B). In contrast, the VAF in the short ccfDNA fraction was consistent up to a family size ≥20 without loss of variant detection in any patient (Fig 6C and 6D). The absolute value of relative percent change in VAF was similar for unselected ccfDNA and short ccfDNA at family sizes ≥5 and ≥10 (Fig 6E). The relative percent change was significantly larger in the unselected ccfDNA compared to the short ccfDNA fraction at a family size ≥15 and a family size ≥20 (Fig 6E). As such, VAF remained more consistent at larger family sizes in the short ccfDNA fraction than in the unselected ccfDNA regardless of initial VAF magnitude. The medium and long ccfDNA fractions exhibited similar improvement in VAF consistency at larger family sizes relative to the unselected ccfDNA (S11 and S12 Figs, respectively), which further supports the strengths of reducing sample complexity to improve sensitivity even though each fraction was associated with loss of variant detection in at least one patient by family size ≥20. Thus, the continued detection of low frequency variants at large family sizes in the short ccfDNA fraction may have been supported by the combined effects of increased consensus read depth and variant enrichment for the total number of reads used in this study.

## Discussion

The results described herein support the automated selection of shorter ccfDNA fragments as a multifactorial approach to improve very low frequency ctDNA detection using NGS. Building upon our previously reported findings from lung cancer [9], this study used both ddPCR and NGS to extend to melanoma, colorectal adenocarcinoma, and pancreatic ductal adenocarcinoma the strengths of size selecting for short ccfDNA fragments to enrich for ctDNA. Furthermore, a high-throughput capable automated size selection technology was implemented that substantially improves the potential to translate these findings to broader research and clinical applications. We also found evidence that selection of short ccfDNA fragments enriches for ctDNA through isolation of fragment sizes containing a greater proportion of variant alleles, while concomitantly reducing wild type alleles that are more abundant at longer fragment lengths. Thus, for a given read depth the detection of ctDNA is more likely in the short ccfDNA fraction where the VAF is greatest. Finally, reduction of sample complexity through *a priori* size-based selection of ccfDNA generated larger family sizes for the subsequent *in silico* suppression of PCR and sequencing errors occurring at very low frequency. Importantly, size selection improved error correction through generation of larger family sizes without adversely affecting variant detection in the short ccfDNA fraction. Collectively, these findings identify the isolation of shorter ccfDNA fragments as a methodology to simultaneously improve both sensitivity and specificity of very low frequency ctDNA detection via NGS.

Investigation of ccfDNA size distribution originated in studies of maternal and fetal DNA in the plasma of pregnant women [16]. As later confirmed for ctDNA, fetus-derived ccfDNA showed an increased occurrence of short fragments when compared to maternal ccfDNA with the predominant peak fraction at ~143–146 bp and a noted absence of dinucleosomal units [17, 18]. With a goal of increasing sensitivity of non-invasive prenatal testing, several studies were successful in moderately enriching for the fetal fraction of circulating DNA through preparative size separation by gel-electrophoresis or microsystem [19–21]. However, the latter methodologies limited size selection to fragments <300 bp, effectively eliminating dinucleosomal and larger plasma DNA without achieving enrichment for the 143 bp fetal peak fraction over the 166 bp maternal component. For enrichment of ctDNA, we have previously used polyacrylamide gel electrophoresis to provide high-resolution manual extraction of targeted ccfDNA fractions [9]. Although this approach enriched for ctDNA in a small cohort of patients with lung cancer [9], the methodology was cumbersome which limited scalability and broader use. In the present study, we demonstrate that high-resolution size-based fractionation to isolate sub-nucleosomal populations is technically feasible using a high-throughput-capable automated gel-extraction platform. In so doing, we also extend our previous observations that selection of short ccfDNA fragments enriches for ctDNA in a broader array of cancer types, including melanoma, colorectal adenocarcinoma, and pancreatic ductal adenocarcinoma. Thus, *a priori* size selection of specific ccfDNA fractions may have greater translational clinical implications for fully harnessing the informational power of ccfDNA size differences in prenatal and cancer diagnostics.

*In silico* analysis is an alternative strategy to the *a priori* physical selection of shorter ccfDNA fragments. The incorporation of *in silico* size analyses of maternal plasma DNA content has facilitated identification of fetal content and diagnosis of fetal aneuploidies [22]. More recently, ccfDNA fragment size has been integrated into *in silico* filtering algorithms to significantly improve the positive predictive value of second-generation prenatal fetal whole genome analysis [23]. However, *in silico* size selection may not enable the potentially advantageous variant allele enrichment afforded by *a priori* physical ccfDNA fragment size selection. In accord, the approach described herein for enrichment may have the greatest use in the search for non-metastatic solid-tumors where ctDNA frequency is commonly <2% [24]. Alternatively, use of *in silico* size selection in combination with physical size selection may further improve ctDNA detection through elimination of the longer fragments observed to migrate with the shorter targeted range. Importantly, isolation of short ccfDNA fractions did not adversely affect VAF by NGS. Although we did observe a greater variance in the calculated gain/loss factor when VAF was determined by ddPCR, this finding was most pronounced for low VAF samples and may be attributable to the amount of library sampled. The ddPCR input of 50 ng comprises only ~1.5–2.5% of the total amount of library. Sampling errors can lead to exaggerated gain/loss results, particularly in low VAF samples where small numbers of under- or over-sampled copies can have dramatic effects on VAF. In contrast, 500 ng (15–25% of total library) were used for hybrid capture and subsequent NGS, likely leading to a more robust estimation of VAF gain/loss factor achieved in each size fraction.

Use of unique molecular identifiers are an increasingly common approach to improve ctDNA specificity. Although duplex molecular barcoding, the assignment of unique molecular identifiers to both strands of DNA, has the greatest theoretical potential to reduce sequencing and PCR errors [25], the associated low ligation efficiency (10–20%) has limited applications seeking to detect very low frequency ctDNA variants due to sample loss [26]. As an alternative, unique molecular identifier performance has been enhanced with error modeling derived from healthy control data substantially reducing false positives, particularly during searches of known variants [11, 26]. Error modeling achieves comparable error reduction as using a barcoded family size ≥ 5 alone [26]. Error modeling may be advantageous as generation of large family sizes has been previously described to lead to a similar loss in sample as duplex molecular barcoding [26]. Here, we showed that *a priori* physical size selection of ccfDNA generated larger family sizes and selection for the short ccfDNA fraction enabled continued successful detection of known variants with a VAF ≥ 0.39% at a family size ≥20 and an average read depth of ~516X. However, using the same sequencing parameters in future investigations may not allow detection at lower VAFs due to the progressive reduction in read depth associated with increments in family size. This was evident in the unselected ccfDNA where the lower VAF, increased sample complexity, and reduced read depth at larger family sizes led to loss of variant detection. Thus, selecting a sufficient read depth for a targeted VAF within the context of using family size data for *in silico* error correction merits strong consideration in future experimental designs. Importantly, our analysis found persistence of stochastic errors even at the largest family sizes with a frequency in the range consistent with very low frequency ctDNA variants (0.1% < VAF < 1%). As such, error modeling alone may not completely eliminate false positives during untargeted searches of ctDNA. Collectively, these observations support the conjecture that unique molecular identifiers, *a priori* physical size selection of short ccfDNA fragments, and sufficient read depth will improve variant detection in early-stage non-metastatic solid tumors or low-frequency aggressive clones in advanced cancers not only through ctDNA enrichment, but also by improving *in silico* error correction through production of larger family sizes.

In addition to size selection of ccfDNA, alternative methods may also improve sensitivity and specificity of ctDNA detection at various steps in the process of generating NGS data. Using a reduced amount of input ccfDNA at library preparation potentially reduces sample complexity and improves generation of larger family sizes. The range of input ccfDNA from patients in this study was 10–56.6 ng (mean: 20.1±14.5 ng) derived from the greater of 10 ng or 1 mL of plasma ccfDNA equivalent. Although we successfully identified variants down to a VAF of 0.39% at a mean read depth of ~5,500X at FS≥1, using less input material may adversely affect sensitivity, particularly for detection of variants with an even lower VAF where using more starting material may be advantageous to minimize type II error due to sampling. Increasing the read depth has the potential to achieve both larger family sizes and greater sensitivity. In this study, a similar number of total reads was used across all samples to evaluate the effects of selecting for subfractions of ccfDNA. Using a larger number of total reads may not achieve a uniform increase in sensitivity and family size between samples as the effect would be less in samples of greater complexity. Thus, future studies may consider evaluating sensitivity and family size within the context of varying sample complexity to determine optimal read depth for a desired VAF. *In silico* analysis using more stringent criteria (i.e., fraction of bases supporting the consensus call, higher quality scores, etc.) may also be an effective approach to reduce false positives. For example, in the present study an alignment score of MQ≥20 and base score Q≥20 with >0.66 concordance between bases was used during consensus identification. Increasing these values may improve specificity, but at the risk of adverse effects on sensitivity. Finally, PCR based methods using molecular barcodes represent a complete alternative approach to the capture-based NGS methods used in this study [27]. While amplicon-based sequencing panels can be useful for querying a small number of mutational hotspots, they rely on consistent amplification of all target sequences in a multiplex PCR step. Therefore, the ability to customize or expand panels by addition of primer pairs is limited. In addition, due to the highly fragmented nature of ccfDNA, amplicon-based approaches can only account for ccfDNA molecules containing intact amplicons, while hybridization probes potentially capture additional unique ccfDNA molecules. Also, hybridization capture sequencing panels can range from small, targeted panels to whole exome or whole genome coverage and size selection has the potential to benefit ctDNA detection using any size panel. Overall, each approach has strengths and weaknesses. A balance between cost, sensitivity, and specificity requires strong consideration when designing a study and determining utility of each method or combination of methods. In this study, we found automated size-based selection of ccfDNA to support both sensitivity and specificity. However, costs associated with labor, equipment, and reagents merit strong consideration prior to integration of size selection for ccfDNA subfractions into a research and clinical laboratory workflow.

Preparation of libraries for physical size selection and NGS requires a multi-step process with multiple rounds of PCR amplification. Our direct comparison of ctDNA VAFs determined by ddPCR in ccfDNA and by multi-step NGS in captured ccfDNA libraries indicated that detectable VAFs were not adversely affected by the NGS methodology used in this study. Although we did observe a reduced association for VAF < 1.5% by ddPCR, this may be attributable to sample size as both the highest (1.31%) and lowest (0.39%) VAF by ddPCR within this subset were increased in the NGS data (2.3% and 0.78%, respectively). A similar comparison of VAFs by sequencing of ccfDNA libraries and by direct ddPCR of the corresponding plasma DNA has been reported [28]. While that report also demonstrated a high correlation of NGS and ddPCR, VAFs in NGS were generally lower (~2X) than those detected directly by ddPCR [28]. We did not observe a similar drift in VAF. This suggests that conversion of ctDNA and non-tumor ccfDNA fragments into NGS libraries may be biased against ctDNA in certain methods of library preparation. Evidence that such bias could at least in part be accounted for by the size difference in non-tumor versus tumor-derived fragments is provided by a previous study which demonstrated that the choice of library preparation method directly influences representation of shorter or damaged ccfDNA molecules [29]. In the present study, however, we did not observe evidence of bias against ctDNA during NGS library preparation. In fact, we found that both WT and variant counts observed by NGS were similar to expected NGS counts using ddPCR data as a reference. The similarities in count number between ddPCR and NGS suggests losses are associated with both approaches. Thus, methods that reduce loss with either technique may further improve overall sensitivity.

Finally, this study has two principal limitations. First, the sample size is relatively small. However, the study intent was not to be a comprehensive clinical study, but rather to determine if the size distribution for ctDNA could be exploited in additional solid tumor malignancies beyond lung cancer. In addition, the cohort size enabled the application of NGS using identical sample preparation and sequencing parameters for buffy coat DNA, unselected ccfDNA, and the short, medium, and long ccfDNA fractions—five samples from each patient—to fully examine the effects of size selection on ctDNA detection. Regardless, future studies would benefit from a larger sample size and continued diversification of human cancers. Second, this study was limited to stage III-IV malignancies and a VAF ≥ 0.39%. Although in some patients the VAF for the known ctDNA variants, the amount of ccfDNA per mL plasma, or both were at a level expected of early-stage lesions, studies specifically examining lower grade cancers are necessary before concluding that size selection has a similarly positive effect on sensitivity and specificity regardless of stage. Similarly, using patient samples with known variants at a lower VAF would be valuable to better appreciate any adverse effects associated with size selection due to material loss when variant alleles are few. Inclusion of samples with a lower VAF would also allow assessment of opposing effects on sensitivity when using larger family sizes for error correction. Nevertheless, the findings from this study provide compelling evidence that investigations using size selection for short ccfDNA fragments to improve ctDNA detection are warranted.

## Methods

### Patient samples and DNA isolation

All human subject research was approved by the University of Utah Institutional Review Board prior to study initiation. Written informed consent was obtained for all study participants under approved IRB protocols #7740, #10924, and #89989. Healthy adult volunteers and cancer patients with a *BRAF* or *KRAS* solid tumor variant associated with a primary melanoma, pancreatic ductal adenocarcinoma, or colorectal adenocarcinoma were recruited for enrollment. Blood samples were collected in BCT tubes (Streck, La Vista, NE) and processed for buffy coat and plasma extraction within 24 hours. The buffy coat and plasma were separated from whole blood by centrifugation at 1,900 g x 10 minutes at 4°C and aspirated to new tubes. Plasma was then centrifuged at 16,000 g x 10 minutes at 4°C to remove any cellular debris. The plasma supernatant and the buffy coat were stored at -80°C until further use. Buffy coat DNA (i.e., white blood cell DNA) was isolated from the buffy coat using the QIAamp DNA Mini Kit (Qiagen, Germantown, MD) and eluted in a final volume of 100 μL 10 mM Tris-Cl and 0.5 mM EDTA (pH 9.0). 100 ng of buffy coat DNA was then sheared using a focused-ultrasonicator (S220, Covaris, Woburn, MA) with a targeted size of 175 bp. Cell-free DNA was isolated from 8 mL of plasma using the QIAamp Circulating Nucleic Acid Kit (Qiagen) and eluted in a final volume of 50 μL 10 mM Tris (pH 8.0) and 0.1 mM EDTA. Cell-free DNA was not sheared.

### NGS library preparation, sequencing, and bioinformatics

Libraries for buffy coat DNA (100 ng) and ccfDNA (10 ng or the quantity equivalent to ccfDNA from 1 mL of plasma, whichever was greater) were prepared using the Kapa Biosystems Hyper Prep Kit for end repair, A-tailing, and ligation of truncated custom IDT adapters that contained an eight base-pair random barcode (i.e., unique molecular identifier) in the index 2 position of a standard Illumina adapter. The Kapa HiFi 2x master mix with truncated-length adapter primer was used for initial library amplification followed by the use of full-length indexing primers during subsequent PCR amplification steps. Full-length buffy coat DNA and ccfDNA libraries were enriched for regions of interest using a custom designed IDT Xgen capture probe set (Integrated DNA Technologies) containing full exonic or hotspot coverage of 128 genes (128 kb; S3 Table). Paired-end sequencing (2x125 bp) of libraries was performed on an Illumina HiSeq 2500. Reads in FASTQ files were aligned to the GRCh37 reference genome and those with the same unclipped alignment start position were grouped into families based on >0.875 molecular barcode similarity. Read sequence was extracted from each family and consensus called on each base position. Those with >0.66 concordance were assigned the predominant base, otherwise, an *N*. See the consensus aligned workflow for a description of the applications [30], settings, and steps taken to generate the consensus alignments (S13 Fig; S1 and S2 Files). Fragment length was derived from paired-end alignment information according to SAM format. Identification of wild type vs. variant allele was determined by a 100% match to an 11 bp string within aligned consensus sequences at the location corresponding to each known variant (S4 Table). Lastly, aligned base error rates and occurrence of localized false positive variants were calculated using our open source EstimateErrorRates and MpileupParser applications available in the USeq package [30]. The USeq EstimateErrorRates application calculates base level error rates observed in quality alignments (≥MQ20) from normal germline sequencing datasets. It parses a Samtools mpileup alignment stack for regions of 7 adjacent bases with adequate read depth (≥100 Q20 bases), no observed indels, and no indication of heterozygous or homozygous SNVs (allele frequencies ≤0.1). Good quality (≥Q20), non-reference, center base observations in each passing region are tabulated. These are used to calculate error rates for each base as well as the total error observed from quality alignments and quality bases. The USeq MpileupParser works in a similar fashion by parsing a Samtools mpileup alignment stack covering bases in a bed file of the 128 kb capture panel coverage with 25 base pair padding. Only quality alignments (≥MQ20) and quality bases (≥Q20) are counted. Locations with evidence of a heterozygous or homozygous allele (AF > 0.1) are ignored. It outputs a bed file of each passing base with its observed non-reference allele frequencies. At FS ≥ 1, allele frequencies were binned (<0.1%, 0.1% to 1.0%, and 1% to 2.0%) and then tracked for presence/absence at subsequent family sizes.

### Fragment size selection

Selection of fractions from truncated ccfDNA libraries was done with an automated liquid handler (NIMBUS Select, Hamilton, Reno, NV) that incorporated Ranger Technology (Coastal Genomics, Burnaby, BC) [31] for the monitoring and real-time manipulation of electrophoretic mobilities through a 3.0% agarose matrix in a 12-channel cassette. Prior to use on human samples, extraction parameters were optimized with a four-rung ladder constructed from lambda phage using Hot Start Taq DNA polymerase (Roche, New York, NY) and the following primer pairs to generate specific lengths of lambda DNA:

278 bp: 5’-GATGCGATGTTATCGGTGCG-3’ and 5’-CACAGGTGAGCCGTGTAGTT-3’
268 bp: 5’-TGGAACCCACCGAGTGAAAG-3’ and 5’-CAATGCAGCAGCAGTCATCC-3’
233 bp: 5’-CGGCACGATCTCGTCAAAAC-3’ and 5’-GCCTTGAACTGAAATGCCCG-3’
223 bp: 5’-GGAAGCTGCATGATGCGATG-3’ and 5’-CTGGTGCGTTTCGTTGGAAG-3’

Ladder lengths were constructed to guide targeting of desired ccfDNA fragment lengths after the addition of the truncated adapters (~103 bp; Fig 1A). The short fraction was optimized to extract a ccfDNA fraction that included both the 223 and 233 bands and no portion of the 268 band, while the long fraction was optimized to include both the 268 and 278 bands, but not the 233 band. After optimization, PCR-amplified truncated ccfDNA libraries (1 μg; Fig 1A) were loaded into the cassette (Coastal Genomics) and short and long fractions were collected from a single run. A second run using intermediate parameters to collect a medium fraction between the short and long fractions from PCR-amplified truncated ccfDNA libraries (1 μg) was also performed. Collected fractions were mixed with QG buffer (Qiagen; 1.4 volumes to 1 volume of sample) and loaded onto a QIAquick spin column from the QIAquick PCR Purification Kit (Qiagen). The remaining manufacturer’s instructions for the kit were then followed and ccfDNA library fractions were eluted in 30 μL of EB buffer (Qiagen). From the eluate, 20 μL was used with full-length indexing primers during PCR amplification in preparation for sequencing (Fig 1A). Densitometry (TapeStation 2200, Agilent Technologies) was used to characterize ccfDNA fragment distribution from unselected and size-selected ccfDNA full-length libraries at a loading concentration 5 ng/μL.

### Droplet digital PCR

Droplet digital PCR (ddPCR) assays were performed on the RainDrop Plus^™^ Digital PCR System (Bio-Rad). For detection of *EGFR* T790M and *KRAS* G13D published assays were used [32, 33]. For additional assays, primer pairs were designed with a target amplicon size <100 bp to accommodate amplification from cell-free DNA samples. Dual-color (FAM/TET) hydrolysis probes containing locked nucleic acid (LNA) nucleotides, 3’ terminal and/or internal quenchers (Iowa Black/ZEN) were designed to distinguish wildtype from mutant alleles. All primers and probes were sourced from Integrated DNA Technologies. Sequences are listed in S5 Table. Reactions were set up in a final volume of 25 μL using TaqMan Genotyping Master Mix (Life Technologies). Primers were added to a final concentration of 500 nM, probes to final concentrations of 100 nM (*BRAF*) or 200nM (all other assays). Up to 10.5 μL of template DNA was tested containing 50 ng of amplified sequencing libraries or varying amounts of cell-free DNA (range: 7–46 ng). False positive noise and limit of blank (LOB) of all assays was determined from a collection of wild-type-only samples and no-template controls (S14 Fig). Data were analyzed using RD Analyst software. In accordance with dMIQE guidelines [34], additional information is provided in S6 Table.

### Size selection of synthetically spiked ccfDNA

Synthetic DNA gBlocks^®^ consisting of 130 bp of genomic *EGFR* sequence spanning the c.2369C>T (p.T790M) point mutation and 165 bp of genomic *BRAF* sequence spanning the c.1799T>A (p.V600E) mutation were purchased from Integrated DNA Technologies. gBlocks^®^ were reconstituted in TE buffer, serially diluted and quantified by ddPCR to determine absolute copy number. Sufficient 130 bp *EGFR* T790M and 165 bp *BRAF* V600E gBlocks^®^ were spiked into a sample of pooled cell-free DNA collected from healthy donors to yield a target VAF of ~10% for both alleles. 10 ng of spiked cell-free DNA and 50 ng of the corresponding unspiked pooled ccfDNA were used for NGS truncated library preparation as described above. The presence of synthetic mutations in the spiked library were verified by ddPCR (*EGFR* T790M 11.4% VAF; *BRAF* V600E 12.1%). Truncated-length libraries of spiked and unspiked samples were subsequently mixed to generate an eight-step serial dilution series. Two independent dilution series were produced. 1 μg of each dilution and unspiked control libraries were size-selected for isolation of short and long fractions as described above. Full-length libraries were produced from extracted fractions and unselected samples and analyzed for *EGFR* T790M and *BRAF* V600E VAF by ddPCR.

### Statistics

For paired samples, the paired t-test was applied. The independent t-test was used for comparison of two independent samples and Levene’s test for inequality determined equal or unequal variance. For multiple samples, one-way analysis of variance (ANOVA) was applied followed by a Tukey post-hoc test for comparisons between pairs of samples. Pearson’s correlation coefficient (*r*) evaluated associations between samples. Boxplots show the median value and the 25^th^ and 75^th^ quartiles. Whiskers on boxplots identify the 5^th^ and the 95^th^ percentiles. For comparison of VAF between different family sizes, the absolute value of relative percent change was calculated to weight all changes in VAF similarly. For comparison of WT and variant counts between ddPCR and NGS, the percent change relative to ddPCR was calculated to normalize the data to account for differences in counts between samples. All statistical analysis was performed in SPSS (Version 24, IBM). Statistical significance was defined as *P* < 0.05.

## Supporting information

S1 Fig Detection of variant alleles by ddPCR in ccfDNA. Plasma ccfDNA was isolated from 13 cancer patients with confirmed solid tumor variants in *BRAF* or *KRAS* (Table 1). Between 7 and 46 ng of ccfDNA was analyzed, depending on the concentration of cell-free DNA in the plasma. Positive control samples were generated from commercial standards (Horizon Discovery; HD701: *BRAF* V600E, *KRAS* G13D; HD239: *BRAF* V600K; HD272: *KRAS* G12D; HD289: *KRAS* G12V) and sheared to mimic cell-free DNA size distribution. Cell-free DNA extracted from the plasma of healthy controls and water were included as wildtype-only and no-template assay controls, respectively. Primary ddPCR data plots and gated areas generated by the RD Analyst software are shown. Gates for wildtype and variant droplet clusters were set using positive control samples and subsequently applied to negative controls and patient samples. The shown variant allele frequency was calculated from the observed droplet counts. Variant copy number per milliliter plasma was extrapolated based on effective analyzed reaction volume, DNA input volume, total extract volume and total plasma volume extracted.
(TIF)

S2 Fig Wild type (WT) and variant allele (VA) counts by ddPCR and NGS. The WT (A) and VA (B) counts are simliar between ddPCR and NGS. In both (A) and (B), the solid line is the line of unity. In (B), the inset is a magnification identified by the box. The legend identifies counts associated with each variant.
(TIF)

S3 Fig Beeswarm plots for insert sizes <250 bp associated with the wild type (WT) and variant allele (VA) from each patient. The solid gray line corresponds to the overall median insert size from all patients of 167 bp. The solid light or dark blue line for WT or VA identifies the corresponding median insert size for that patient. In some instance it is not visible (e.g., M1) as it is behind the gray line. The identifiers under each plot are matched to Table 1 and Fig 1C. C = colorectal adenocarcinoma; M = melanoma; P = pancreatic ductal adenocarcinoma.
(TIF)

S4 Fig Beeswarm plots for insert sizes >250 bp associated with the wild type (WT) and variant allele (VA) from each patient. Absence of data (e.g., C1) indicates that an allele with an insert size > 250 bp was not detected for that patient. The solid black line for WT or VA identifies the median insert size. The dark blue and light blue numbers identify the total number of counts for WT and VA, respectively. The identifiers under each plot are matched to Table 1 and Fig 1C. The percentages under each identifier indicate VAF using insert sizes >250 bp. C = colorectal adenocarcinoma; M = melanoma; P = pancreatic ductal adenocarcinoma.
(TIF)

S5 Fig VAFs detected by ddPCR of size-selected and unselected synthetically spiked ccfDNA libraries. Pooled normal ccfDNA was spiked with 130-bp *EGFR* T790M and 165-bp *BRAF* V600E synthetic gBlocks^®^ and truncated libraries were prepared from the spiked sample and its unspiked reference pool. After creation of an eight-step dilution series of spiked with unspiked controls, the spiked libraries and unspiked reference were size selected. Full-length libraries were subsequently prepared from unselected samples and short and long gel fractions. VAF for 130-bp *EGFR* T790M and 165-bp *BRAF* V600E was detected by ddPCR using 50 ng of full-length library.
(TIF)

S6 Fig VAF by ddPCR in size-selected ccfDNA libraries. Full-length libraries were prepared from unselected samples and short, medium, and long fractions. VAF of known variant was determined by ddPCR from 50 ng of library. Primary ddPCR data plots and gated areas generated by the RD Analyst software are shown. Gates for wildtype and variant droplet clusters were set on the unselected sample and applied to patient-matched size selected samples.
(TIF)

S7 Fig Percent difference in wild type (WT) and variant counts for each ccfDNA fraction relative to unselected ccfDNA counts. Compared to WT counts in unselected ccfDNA, there was a significant reduction in the short ccfDNA fraction compared to the medium and long ccfDNA fractions (A). For the variant counts (B), there was a significant reduction in the long ccfDNA fraction compared to the medium ccfDNA fraction and a strong trend to have fewer counts than the short ccfDNA fraction. *P<0.05, **P<0.01, ***P<0.001.
(TIF)

S8 Fig Median insert size for the wild type (WT) and variant allele (VA) for each ccfDNA fraction. Within each subfraction of the mononucleosome, there was evidence that the VA was shorter and had a broader distribution of insert sizes than the WT allele.
(TIF)

S9 Fig Generation of family sizes in buffy coat DNA, unselected ccfDNA, short, medium and long ccfDNA fractions. Overall, total reads were similar between sample types except for the long ccfDNA fraction where there was a significant increase (A). Consensus read depth (family size ≥1) was greatest in buffy coat DNA and least in the short ccfDNA fraction (B). The on-target fraction was similar across all sample types except for the short ccfDNA fraction where there was a significant decrease (C). Average family size was greatest in the short ccfDNA, while the family sizes in the medium and long fractions were significantly larger than the buffy coat DNA (D). At the specific variant locations for each patient, consensus read depth at family size ≥20 was greatest in the short, medium, and long fraction (E). In (A-E), solid bars represent the mean value and whiskers correspond to the standard deviation. *** *P* ≤0.001; ** *P* = 0.01; * *P* < 0.05; NS = not significant.
(TIF)

S10 Fig Comparison of coverage, on-target fraction, and family size between unselected ccfDNA from healthy controls and patients. Although total reads (A), consensus read depth (B), and on-target fraction (C) were significantly higher in the patient cohort, the average family size was largest in the controls (D). In (A-D), solid bars represent the mean value and whiskers correspond to the standard deviation.
(TIF)

S11 Fig Effects of family size on VAF in the medium ccfDNA fraction. Overall, VAF was relatively stable up to a family size ≥15 in the medium fraction of ccfDNA (A). However, at larger family sizes VAF became less stable and included complete loss of variants in some samples (B, magnification of area in blue box shown in A). The relative percent difference in VAF was similar in unselected and medium ccfDNA at family size (FS) ≥5 and FS ≥10, but was significantly larger in unselected ccfDNA at FS ≥15 (C). At FS ≥20, there was a trend for a larger difference of VAF in the unselected ccfDNA, but it was not statistically significant. *P < 0.05; NS = not significant.
(TIF)

S12 Fig Effects of family size on VAF in the long ccfDNA fraction. Overall, VAF was relatively stable in the long ccfDNA fraction (A) even at large family sizes and lowest VAFs (B, magnification of area in blue box shown in A). Of note, in one sample the variant allele was lost at FS ≥6. The relative percent difference in VAF was similar in unselected and long ccfDNA at family size (FS) ≥5, FS ≥10, and FS ≥15, but was significantly larger in unselected ccfDNA at FS ≥20 (C). *** *P* ≤ 0.001; NS = not significant.
(TIF)

S13 Fig Consensus alignment workflow. A Snakemake workflow was constructed to convert fastq sequencing datasets with unique molecular identifiers to processed alignments. This involved 18 steps as represented in this directed acyclic graph. In brief, alignments are generated with bwa. Those with the same unclipped start position are grouped by UMI and collapsed to a single error corrected consensus sequence with USeq tools. These are aligned, merged, and passed through GATK’s best practice INDEL realignment and base score recalibration process. Throughout, various quality control files are generated including a unique observation read coverage data track. See the snakemake file (S1 File) and bash script file (S2 File) for exact program versions and parameter settings.
(TIF)

S14 Fig False positive droplet events in control samples. For each ddPCR assay false positive droplet events were measured in a collection of controls (A). Samples tested in each assay included full-length libraries (n≥11), plasma cell-free DNA (n≥9), buffy coat DNA (n≤3) and no template controls (n≤3). A Poisson model was applied to fit the observed false positive distribution (dashed line). The mean of the Poisson distribution (λ) was determined and the limit of blank (LOB) for each assay was calculated from the 95% confidence interval of the Poisson distribution as well as from the 95% limit of the empirical distribution. False positive variant allele frequency (VAF) was determined for each control experiment, excluding no template controls (B). Median VAF, interquartile range and 95 percentile (error bars) of false positive VAFs for each assay are indicated. Data are summarized in table format (C).
(TIF)

S1 Table ccfDNA concentration, unique copies of wild type (WT) and variant alleles (VA) by ddPCR and sequencing, and variant allele frequency (VAF). (DOCX)

S2 Table Counts of wild type (WT) and variant alleles (VA) by NGS* and variant allele frequency (VAF) for unselected ccfDNA and the size-selected fractions. (DOCX)

S3 Table 128 genes included in next generation capture panel. (DOCX)

S4 Table Eleven base-pair strings used to differentiate wild type alleles from variant alleles at allele specific locations. (DOCX)

S5 Table ddPCR primers and probes. (DOCX)

S6 Table Additional essential ddPCR metrics as required according to the digital MIQE guidelines (Ref. 34). Data provided summarize all ddPCR experiments performed as part of this study.
(DOCX)

S1 File Snakemake rule set for executing the consensus alignment workflow. (SM)

S2 File Example slurm cluster scheduler bash script for executing the consensus alignment workflow. (SH)

S3 File Data for all figures. (XLSX)
