# Supplementary figures and images for: Automated size selection for short cell-free DNA fragments enriches for circulating tumor DNA and improves error correction during next generation sequencing
Source: PLoS One. 2018 Jul 25;13(7):e0197333. doi: 10.1371/journal.pone.0197333 (PMC6059400; doi:10.1371/journal.pone.0197333)

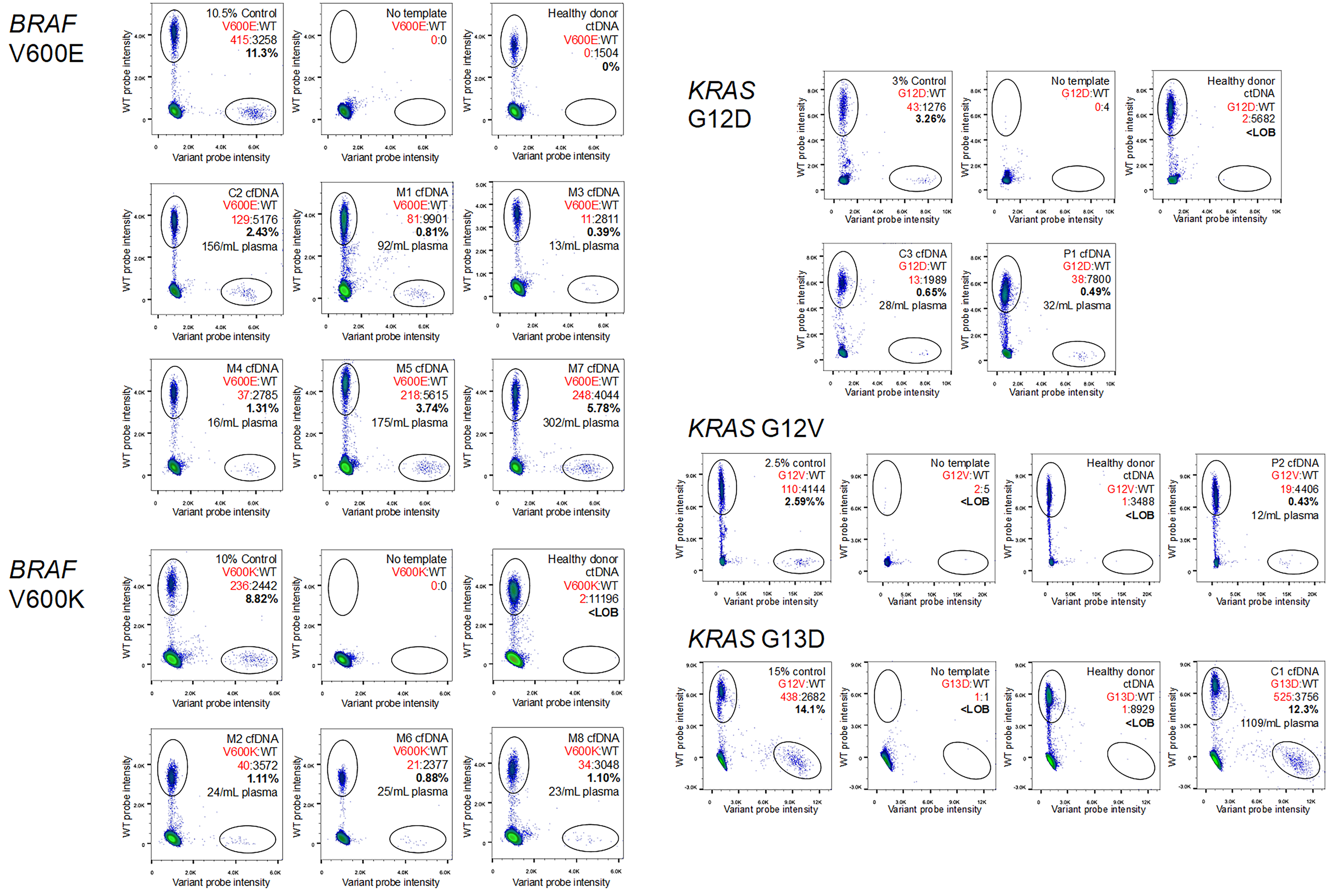

Supplement: S1 Fig — Plasma ccfDNA was isolated from 13 cancer patients with confirmed solid tumor variants in BRAF or KRAS (Table 1). Between 7 and 46 ng of ccfDNA was analyzed, depending on the concentration of cell-free DNA in the plasma. Positive control samples were generated from commercial standards (Horizon Discovery; HD701: BRAF V600E, KRAS G13D; HD239: BRAF V600K; HD272: KRAS G12D; HD289: KRAS G12V) and sheared to mimic cell-free DNA size distribution. Cell-free DNA extracted from the plasma of healthy controls and water were included as wildtype-only and no-template assay controls, respectively. Primary ddPCR data plots and gated areas generated by the RD Analyst software are shown. Gates for wildtype and variant droplet clusters were set using positive control samples and subsequently applied to negative controls and patient samples. The shown variant allele frequency was calculated from the observed droplet counts. Variant copy number per milliliter plasma was extrapolated based on effective analyzed reaction volume, DNA input volume, total extract volume and total plasma volume extracted. (TIF) [file pone.0197333.s001.tif]

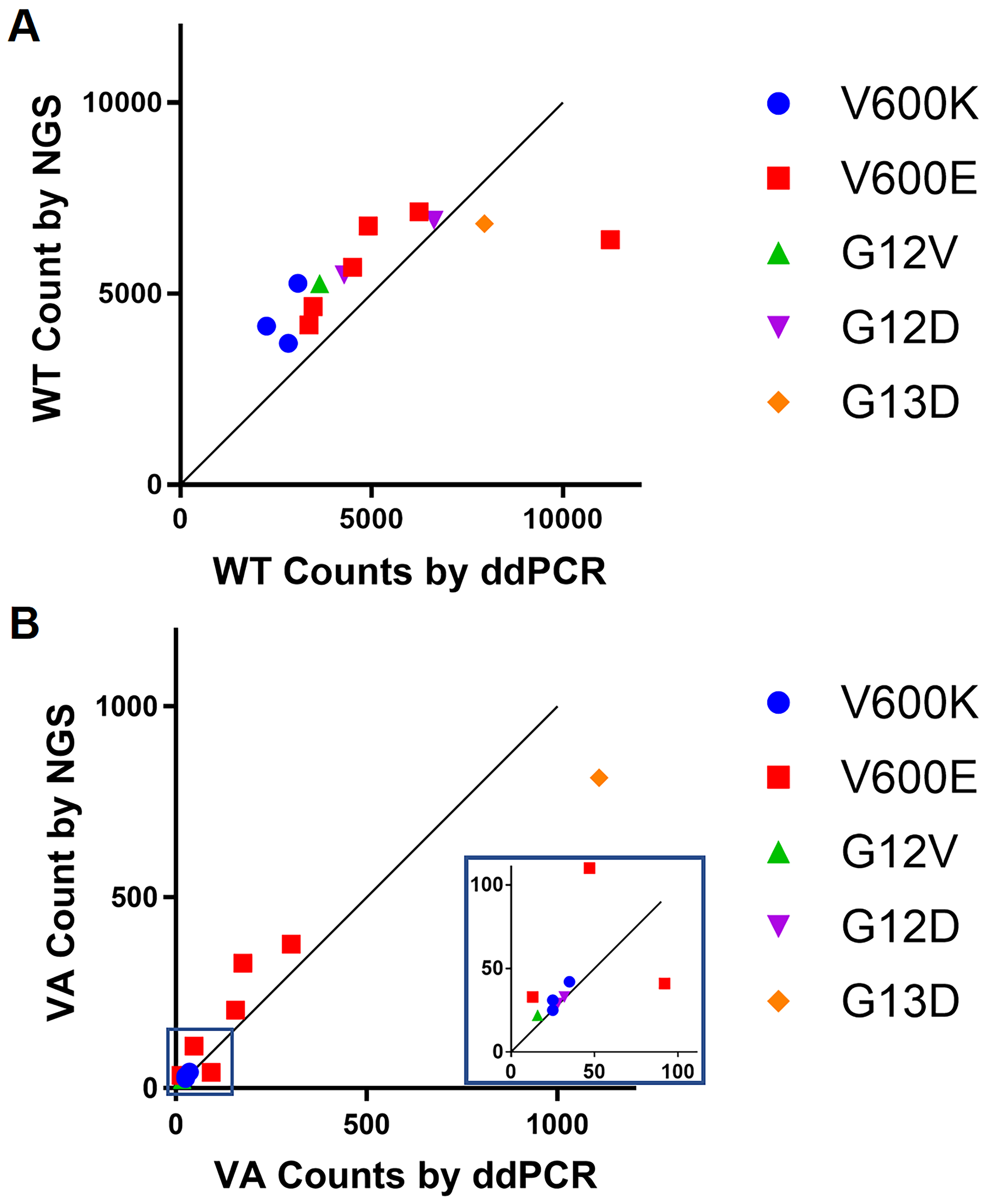

Supplement: S2 Fig — The WT (A) and VA (B) counts are simliar between ddPCR and NGS. In both (A) and (B), the solid line is the line of unity. In (B), the inset is a magnification identified by the box. The legend identifies counts associated with each variant. (TIF) [file pone.0197333.s002.tif]

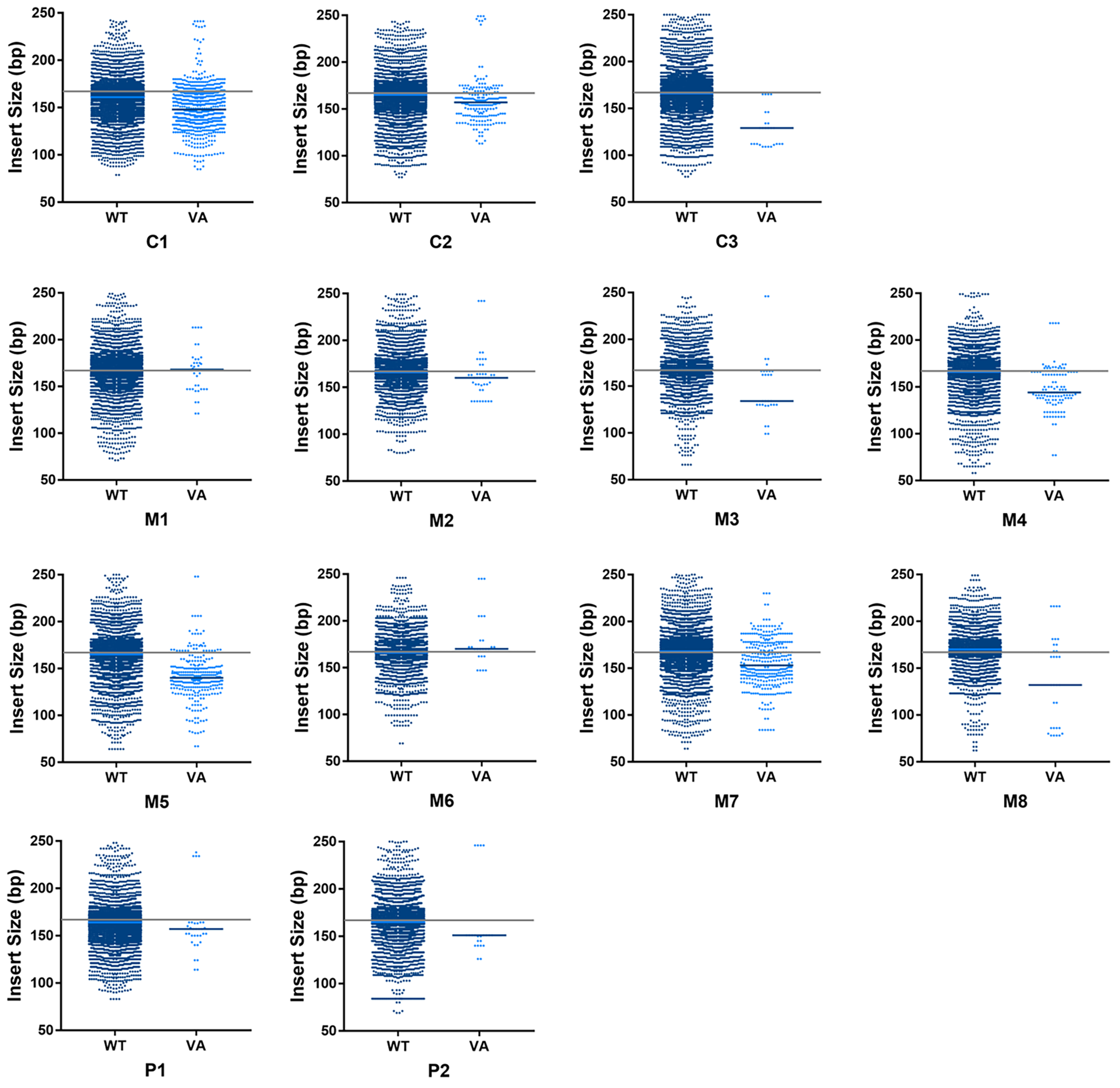

Supplement: S3 Fig — The solid gray line corresponds to the overall median insert size from all patients of 167 bp. The solid light or dark blue line for WT or VA identifies the corresponding median insert size for that patient. In some instance it is not visible (e.g., M1) as it is behind the gray line. The identifiers under each plot are matched to Table 1 and Fig 1C. C = colorectal adenocarcinoma; M = melanoma; P = pancreatic ductal adenocarcinoma. (TIF) [file pone.0197333.s003.tif]

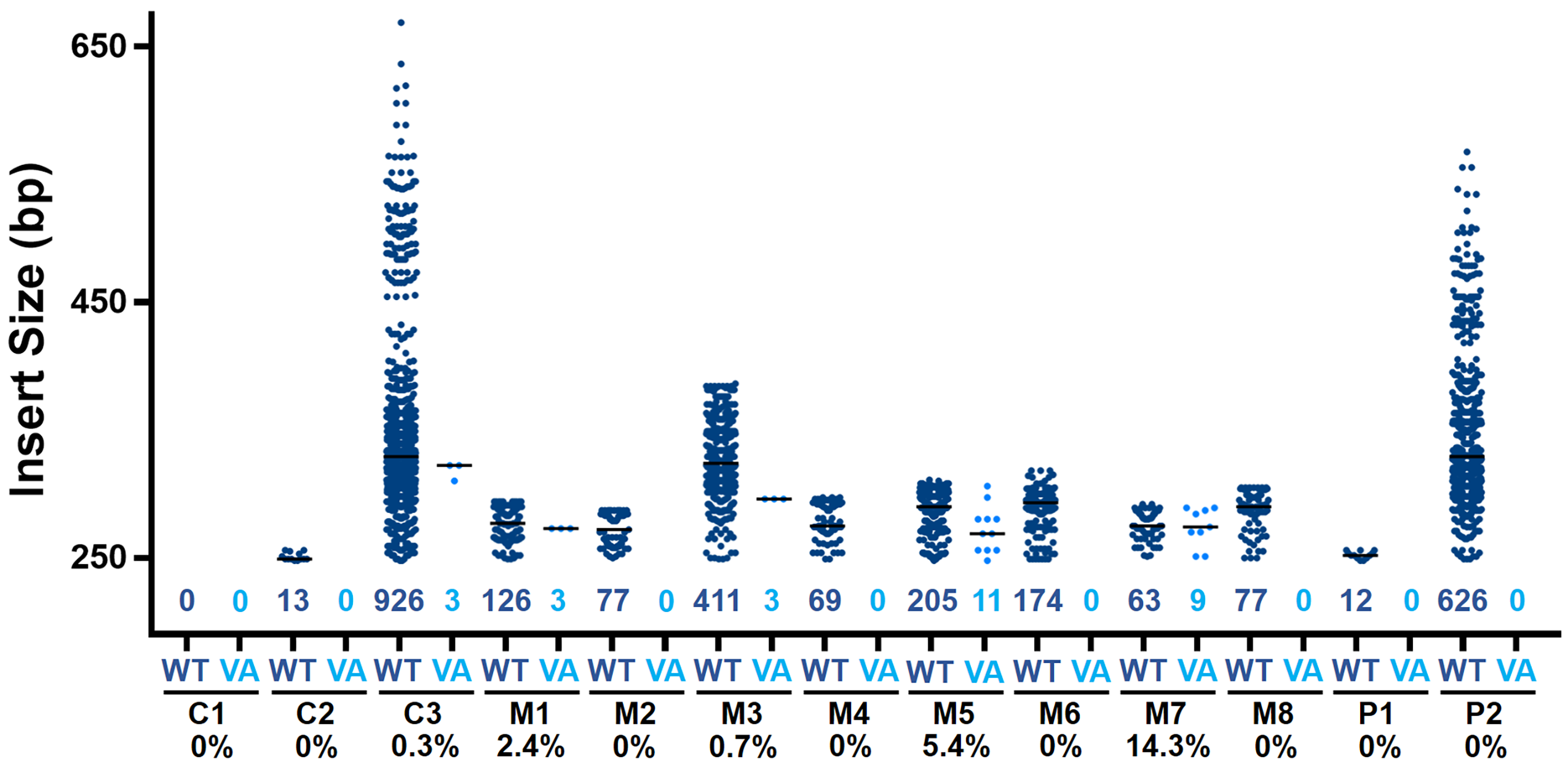

Supplement: S4 Fig — Absence of data (e.g., C1) indicates that an allele with an insert size > 250 bp was not detected for that patient. The solid black line for WT or VA identifies the median insert size. The dark blue and light blue numbers identify the total number of counts for WT and VA, respectively. The identifiers under each plot are matched to Table 1 and Fig 1C. The percentages under each identifier indicate VAF using insert sizes >250 bp. C = colorectal adenocarcinoma; M = melanoma; P = pancreatic ductal adenocarcinoma. (TIF) [file pone.0197333.s004.tif]

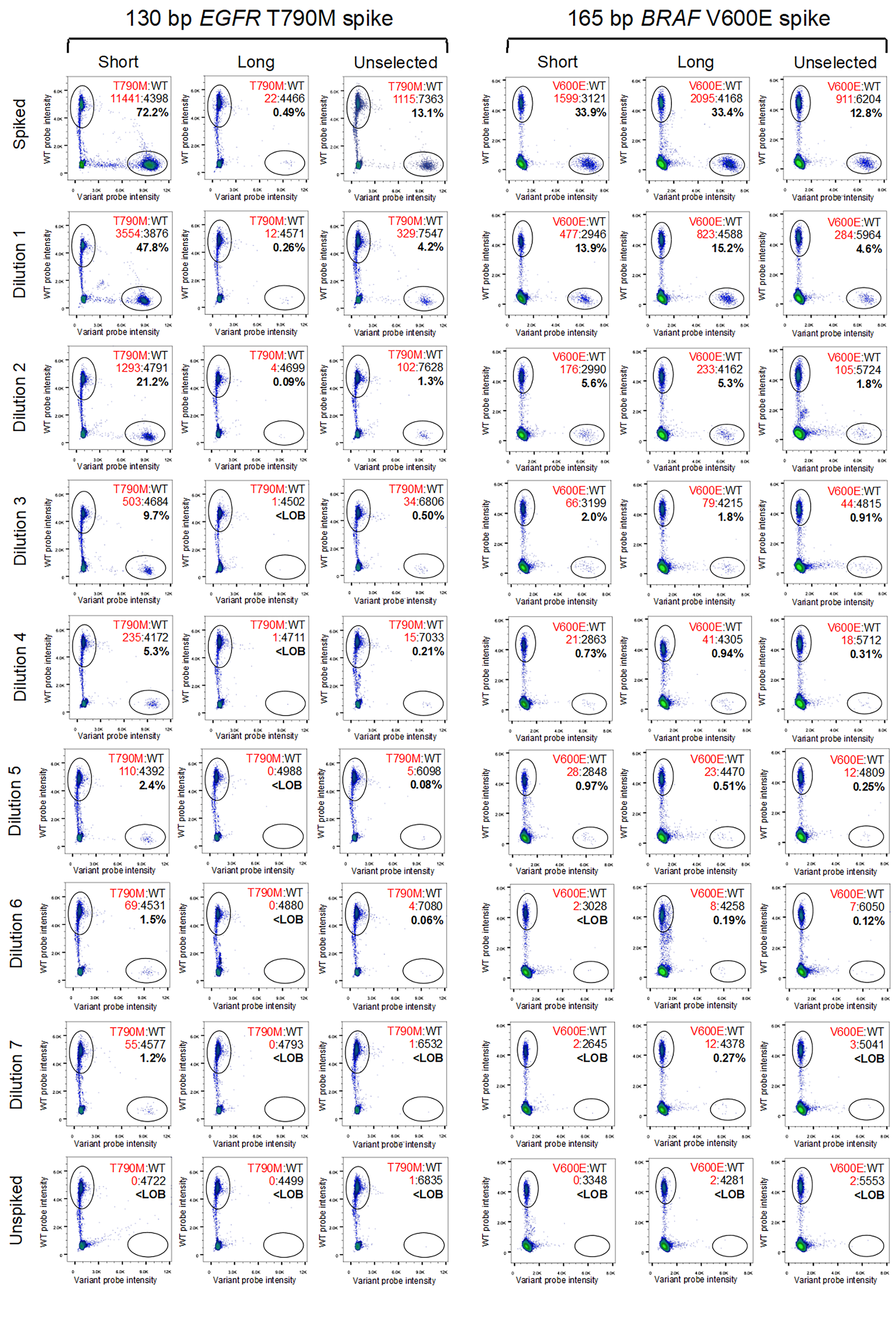

Supplement: S5 Fig — Pooled normal ccfDNA was spiked with 130-bp EGFR T790M and 165-bp BRAF V600E synthetic gBlocks® and truncated libraries were prepared from the spiked sample and its unspiked reference pool. After creation of an eight-step dilution series of spiked with unspiked controls, the spiked libraries and unspiked reference were size selected. Full-length libraries were subsequently prepared from unselected samples and short and long gel fractions. VAF for 130-bp EGFR T790M and 165-bp BRAF V600E was detected by ddPCR using 50 ng of full-length library. (TIF) [file pone.0197333.s005.tif]

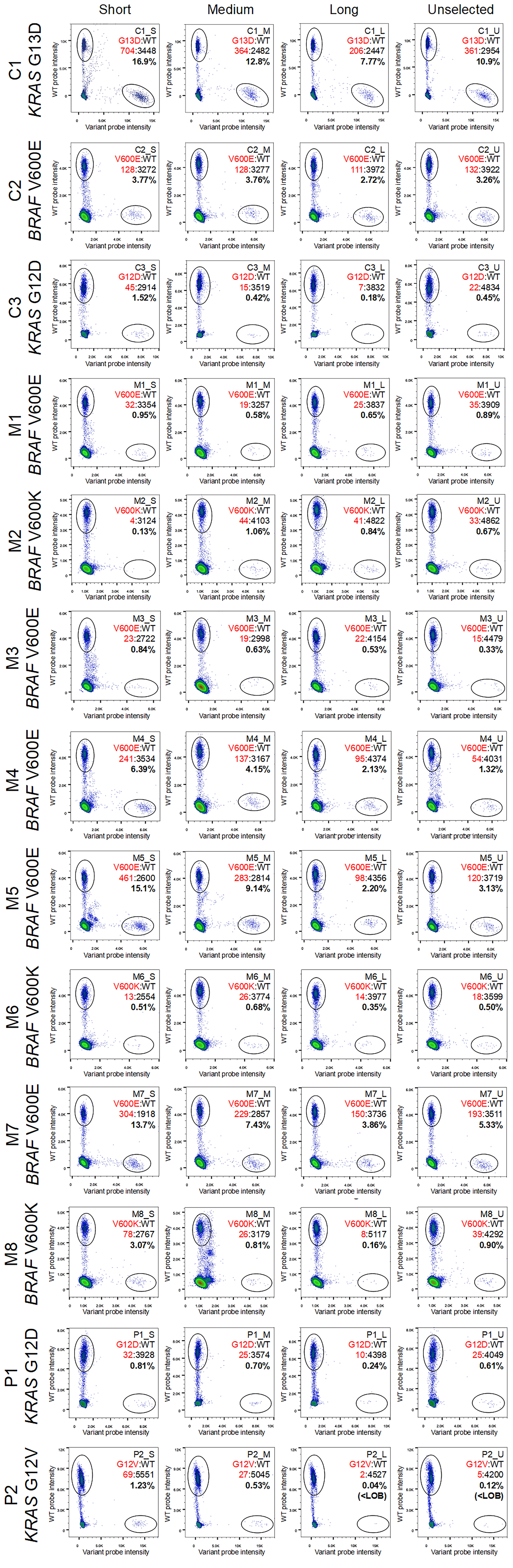

Supplement: S6 Fig — Full-length libraries were prepared from unselected samples and short, medium, and long fractions. VAF of known variant was determined by ddPCR from 50 ng of library. Primary ddPCR data plots and gated areas generated by the RD Analyst software are shown. Gates for wildtype and variant droplet clusters were set on the unselected sample and applied to patient-matched size selected samples. (TIF) [file pone.0197333.s006.tif]

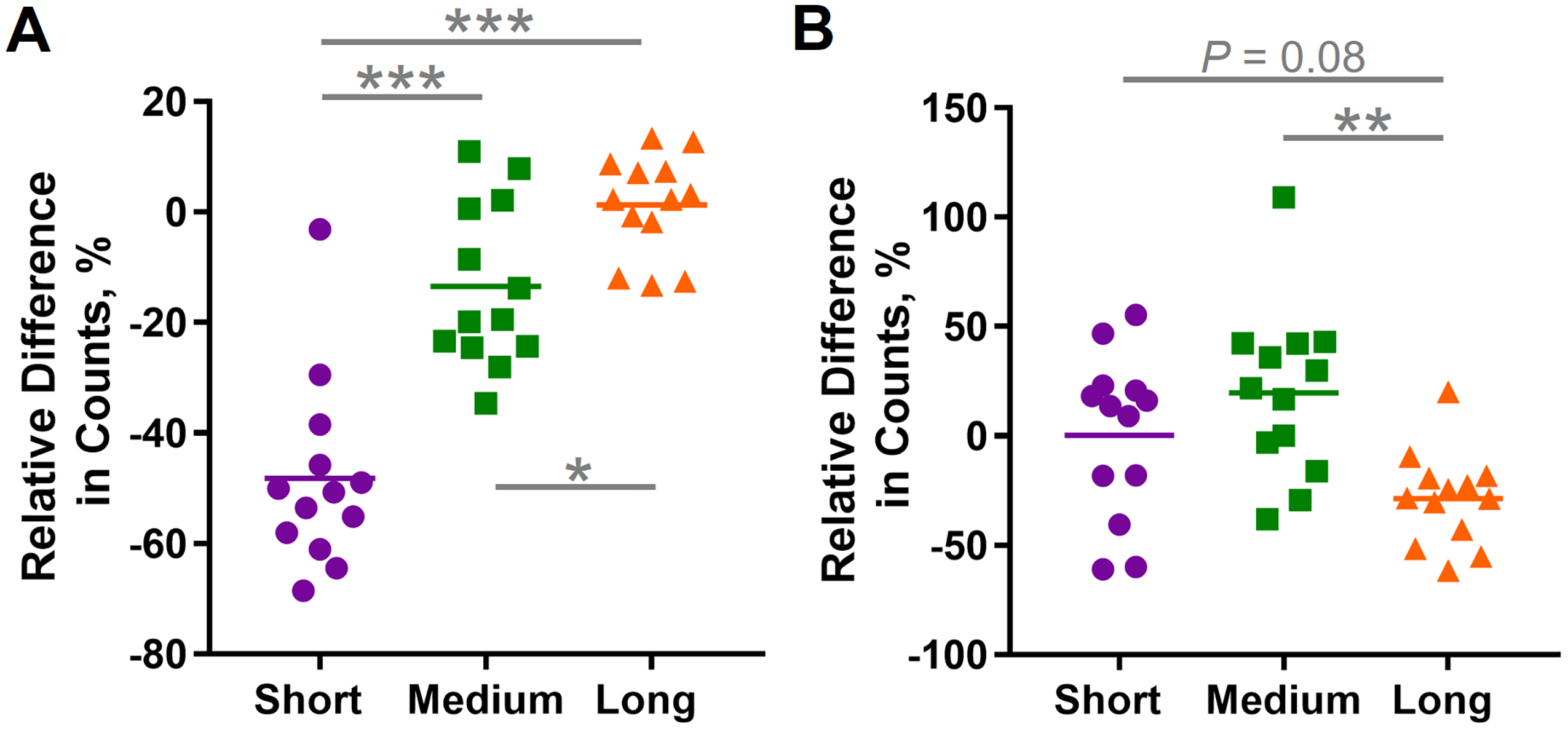

Supplement: S7 Fig — Compared to WT counts in unselected ccfDNA, there was a significant reduction in the short ccfDNA fraction compared to the medium and long ccfDNA fractions (A). For the variant counts (B), there was a significant reduction in the long ccfDNA fraction compared to the medium ccfDNA fraction and a strong trend to have fewer counts than the short ccfDNA fraction. *P<0.05, **P<0.01, ***P<0.001. (TIF) [file pone.0197333.s007.tif]

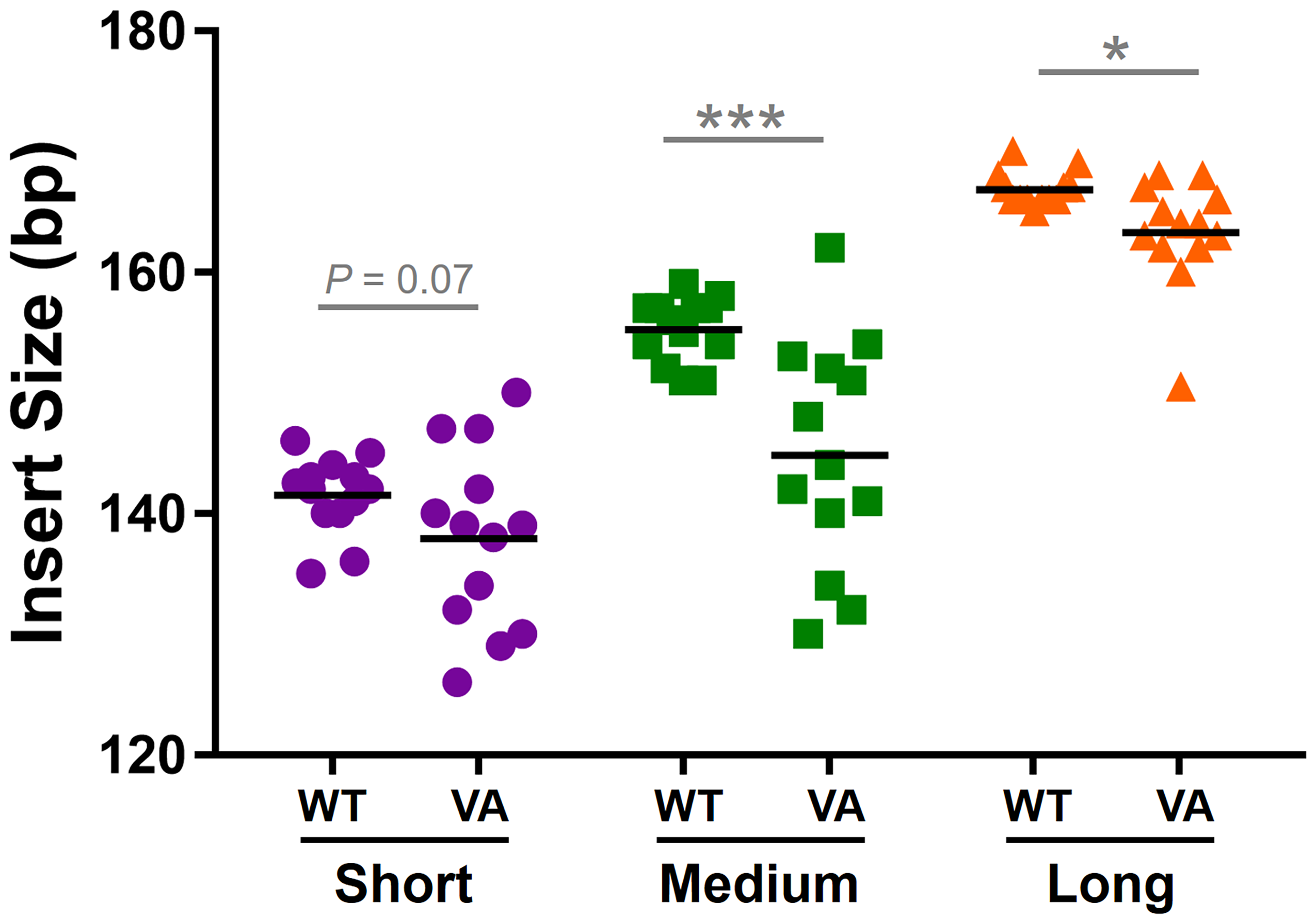

Supplement: S8 Fig — Within each subfraction of the mononucleosome, there was evidence that the VA was shorter and had a broader distribution of insert sizes than the WT allele. (TIF) [file pone.0197333.s008.tif]

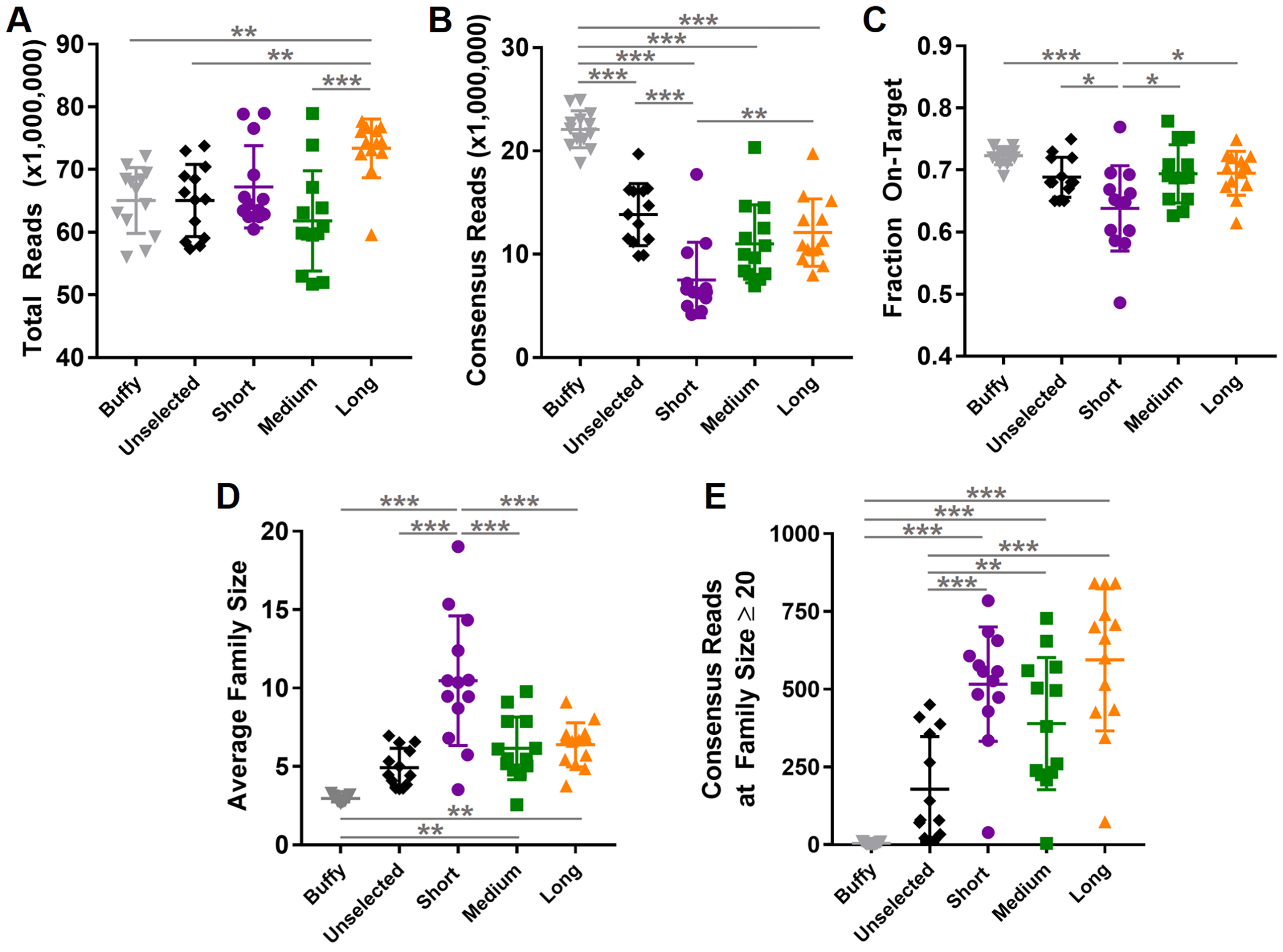

Supplement: S9 Fig — Overall, total reads were similar between sample types except for the long ccfDNA fraction where there was a significant increase (A). Consensus read depth (family size ≥1) was greatest in buffy coat DNA and least in the short ccfDNA fraction (B). The on-target fraction was similar across all sample types except for the short ccfDNA fraction where there was a significant decrease (C). Average family size was greatest in the short ccfDNA, while the family sizes in the medium and long fractions were significantly larger than the buffy coat DNA (D). At the specific variant locations for each patient, consensus read depth at family size ≥20 was greatest in the short, medium, and long fraction (E). In (A-E), solid bars represent the mean value and whiskers correspond to the standard deviation. *** P ≤0.001; ** P = 0.01; * P < 0.05; NS = not significant. (TIF) [file pone.0197333.s009.tif]

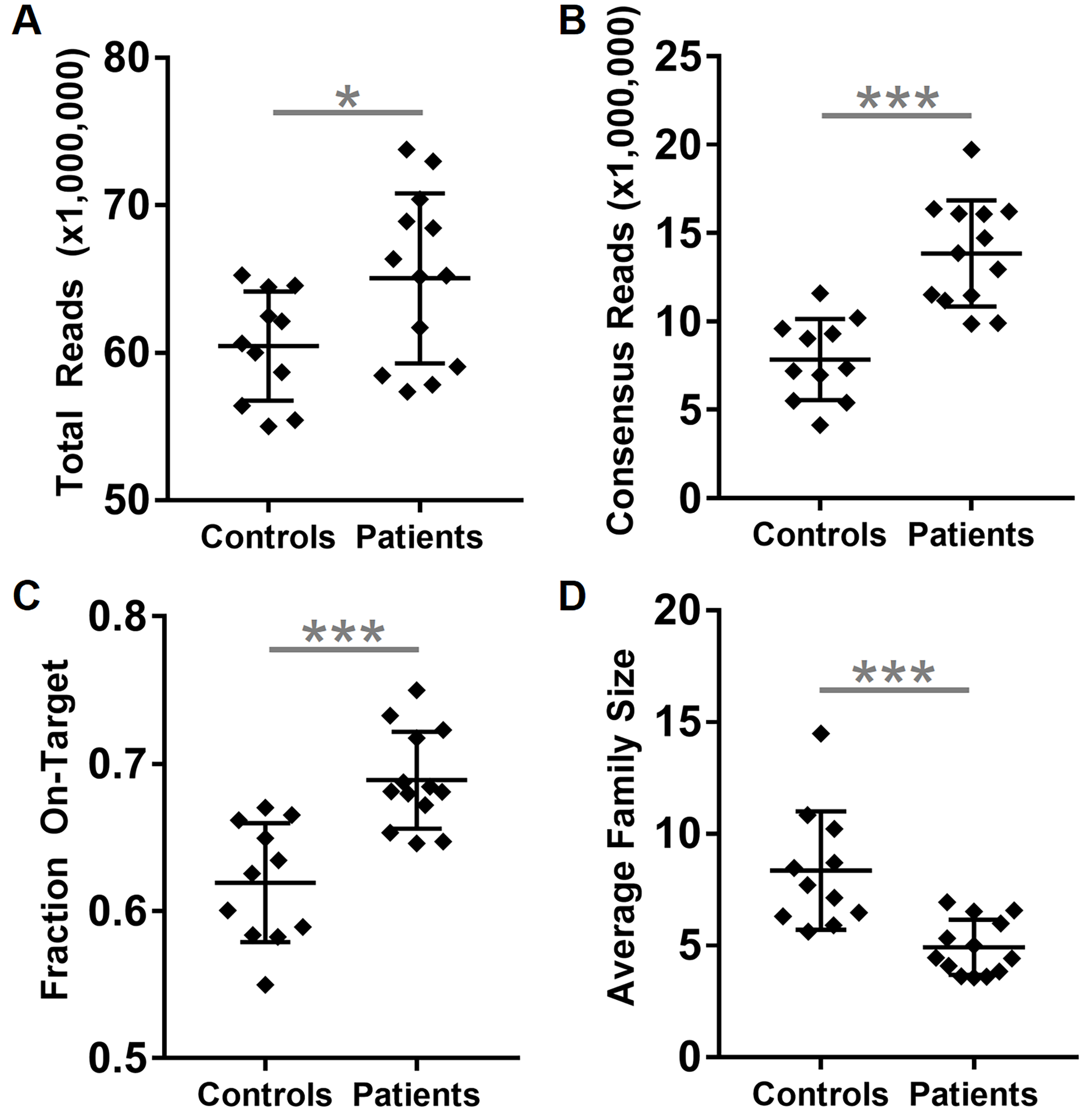

Supplement: S10 Fig — Although total reads (A), consensus read depth (B), and on-target fraction (C) were significantly higher in the patient cohort, the average family size was largest in the controls (D). In (A-D), solid bars represent the mean value and whiskers correspond to the standard deviation. (TIF) [file pone.0197333.s010.tif]

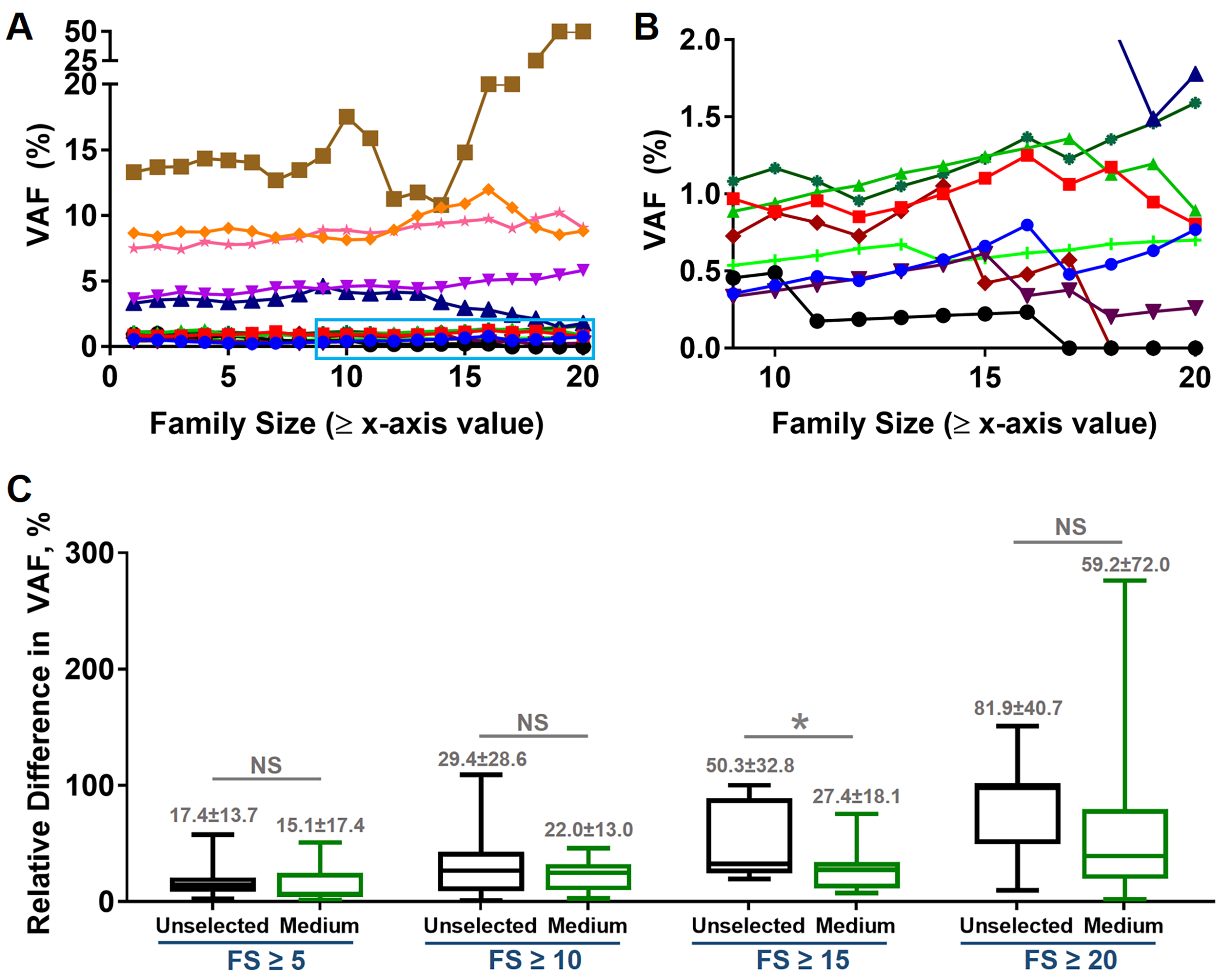

Supplement: S11 Fig — Overall, VAF was relatively stable up to a family size ≥15 in the medium fraction of ccfDNA (A). However, at larger family sizes VAF became less stable and included complete loss of variants in some samples (B, magnification of area in blue box shown in A). The relative percent difference in VAF was similar in unselected and medium ccfDNA at family size (FS) ≥5 and FS ≥10, but was significantly larger in unselected ccfDNA at FS ≥15 (C). At FS ≥20, there was a trend for a larger difference of VAF in the unselected ccfDNA, but it was not statistically significant. *P < 0.05; NS = not significant. (TIF) [file pone.0197333.s011.tif]

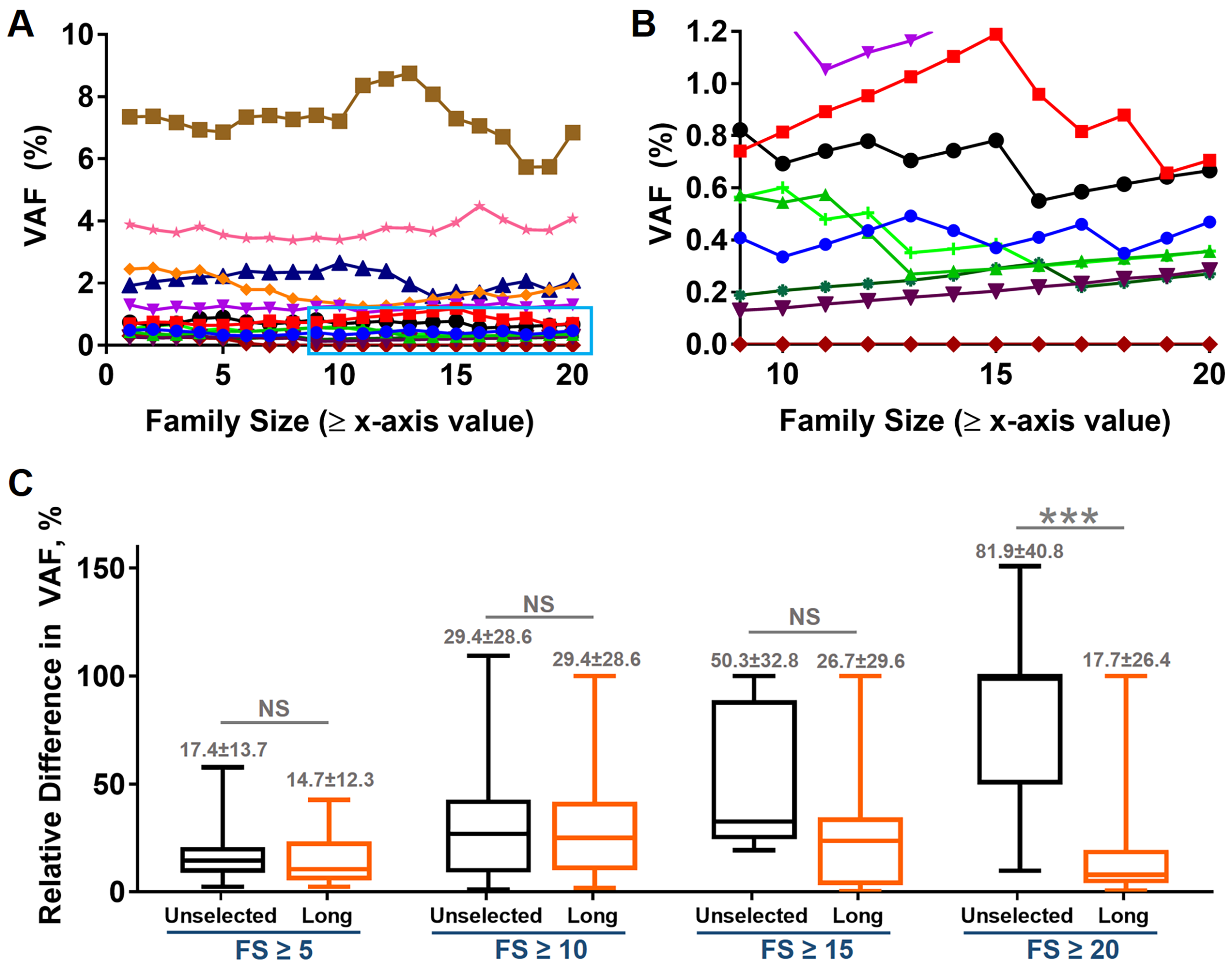

Supplement: S12 Fig — Overall, VAF was relatively stable in the long ccfDNA fraction (A) even at large family sizes and lowest VAFs (B, magnification of area in blue box shown in A). Of note, in one sample the variant allele was lost at FS ≥6. The relative percent difference in VAF was similar in unselected and long ccfDNA at family size (FS) ≥5, FS ≥10, and FS ≥15, but was significantly larger in unselected ccfDNA at FS ≥20 (C). *** P ≤ 0.001; NS = not significant. (TIF) [file pone.0197333.s012.tif]

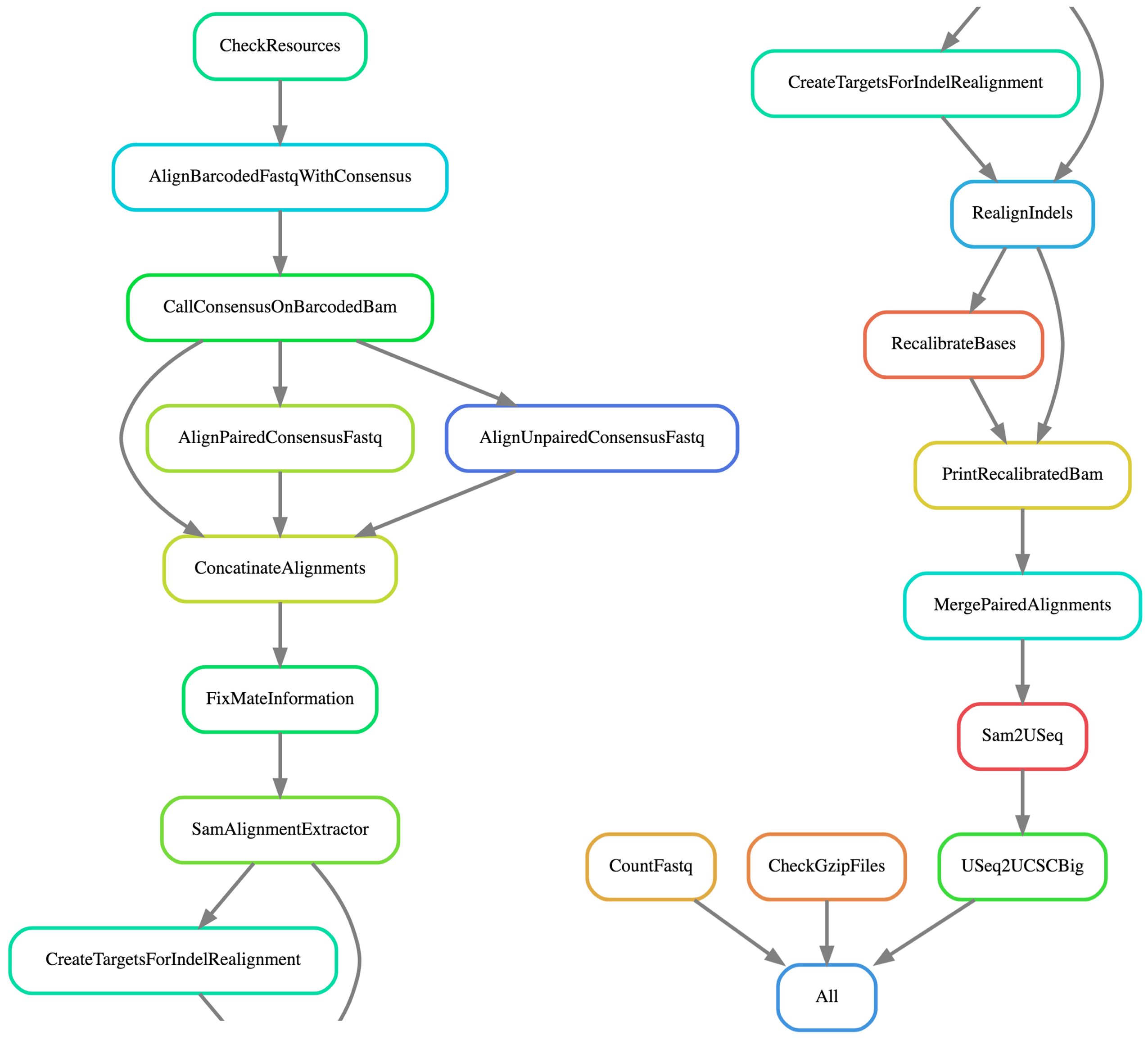

Supplement: S13 Fig — A Snakemake workflow was constructed to convert fastq sequencing datasets with unique molecular identifiers to processed alignments. This involved 18 steps as represented in this directed acyclic graph. In brief, alignments are generated with bwa. Those with the same unclipped start position are grouped by UMI and collapsed to a single error corrected consensus sequence with USeq tools. These are aligned, merged, and passed through GATK’s best practice INDEL realignment and base score recalibration process. Throughout, various quality control files are generated including a unique observation read coverage data track. See the snakemake file (S1 File) and bash script file (S2 File) for exact program versions and parameter settings. (TIF) [file pone.0197333.s013.tif]

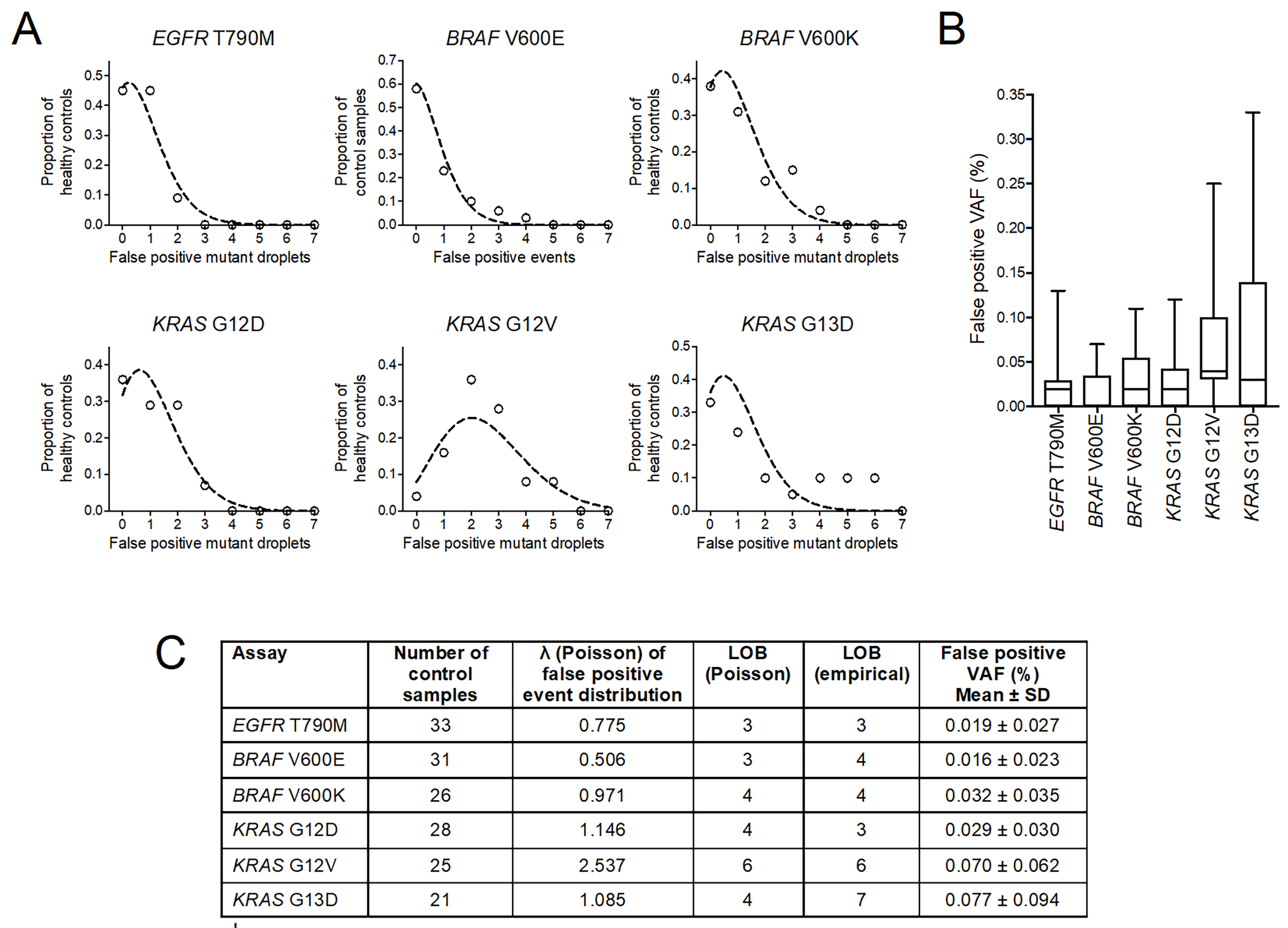

Supplement: S14 Fig — For each ddPCR assay false positive droplet events were measured in a collection of controls (A). Samples tested in each assay included full-length libraries (n≥11), plasma cell-free DNA (n≥9), buffy coat DNA (n≤3) and no template controls (n≤3). A Poisson model was applied to fit the observed false positive distribution (dashed line). The mean of the Poisson distribution (λ) was determined and the limit of blank (LOB) for each assay was calculated from the 95% confidence interval of the Poisson distribution as well as from the 95% limit of the empirical distribution. False positive variant allele frequency (VAF) was determined for each control experiment, excluding no template controls (B). Median VAF, interquartile range and 95 percentile (error bars) of false positive VAFs for each assay are indicated. Data are summarized in table format (C). (TIF) [file pone.0197333.s014.tif]
