# Supplementary material for: Automated size selection for short cell-free DNA fragments enriches for circulating tumor DNA and improves error correction during next generation sequencing
Source: PLoS One. 2018 Jul 25;13(7):e0197333. doi: 10.1371/journal.pone.0197333 (PMC6059400; doi:10.1371/journal.pone.0197333)
Supplement: S1 Table — (DOCX) [file pone.0197333.s015.docx]

**S1 Table. ccfDNA concentration, unique copies of wild type (WT) and variant alleles (VA) by ddPCR and sequencing, and variant allele frequency (VAF).**

| **ID** | **Allele** | **Yield of ccfDNA**  **(ng/mL plasma)** | **ccfDNA input for NGS library prep (ng)** | **ddPCR** | | | **Expected number of copies in NGS library prep by ddPCR*** | | **NGS**** | | |
| --- | --- | --- | --- | --- | --- | --- | --- | --- | --- | --- | --- |
|  |  |  |  | **WT copies** | **VA copies** | **VAF, %** | **WT copies** | **VA copies** | **WT copies** | **VA copies** | **VAF,**  **%** |
| C1 | *KRAS* G13D | 56.6 | 56.6 | 3756 | 525 | 12.26 | 7950 | 1109 | 6836 | 813 | 10.63 |
| C2 | *BRAF* V600E | 24.7 | 24.7 | 5176 | 129 | 2.43 | 6244 | 156 | 7149 | 204 | 2.77 |
| C3 | *KRAS* G12D | 29.7 | 29.7 | 1989 | 13 | 0.65 | 4282 | 28 | 5491 | 29 | 0.53 |
| M1 | *BRAF* V600E | 26.6 | 26.6 | 9901 | 81 | 0.81 | 11247 | 92 | 6410 | 41 | 0.64 |
| M2 | *BRAF* V600K | 6.9 | 10.0 | 3572 | 40 | 1.11 | 3071 | 35 | 5279 | 42 | 0.79 |
| M3 | *BRAF* V600E | 12.7 | 12.7 | 2811 | 11 | 0.39 | 3368 | 13 | 4180 | 33 | 0.78 |
| M4 | *BRAF* V600E | 3.4 | 10.0 | 2785 | 37 | 1.31 | 3478 | 47 | 4666 | 110 | 2.30 |
| M5 | *BRAF* V600E | 13.5 | 13.5 | 5615 | 218 | 3.74 | 4508 | 175 | 5691 | 327 | 5.43 |
| M6 | *BRAF* V600K | 34.2 | 34.2 | 2377 | 21 | 0.88 | 2823 | 25 | 3709 | 25 | 0.67 |
| M7 | *BRAF* V600E | 17.4 | 17.4 | 4044 | 248 | 5.78 | 4918 | 302 | 6777 | 377 | 5.27 |
| M8 | *BRAF* V600K | 9.1 | 10.0 | 3048 | 34 | 1.10 | 2253 | 25 | 4155 | 31 | 0.74 |
| P1 | *KRAS* G12D | 18.6 | 18.6 | 7800 | 38 | 0.48 | 6633 | 32 | 6921 | 33 | 0.47 |
| P2 | *KRAS* G12V | 7.3 | 10.0 | 4406 | 19 | 0.43 | 3645 | 16 | 5264 | 22 | 0.42 |

*Expected copies were extrapolated from the ccfDNA sample tested by ddPCR. Input DNA amount, effectively analyzed reaction volume, and Poisson correction for each ddPCR reaction were taken into account to infer copy numbers in the input amount of cell-free DNA used for each corresponding library prep.

**Unique consensus reads for family size ≥1
