# Supplementary material for: Automated size selection for short cell-free DNA fragments enriches for circulating tumor DNA and improves error correction during next generation sequencing
Source: PLoS One. 2018 Jul 25;13(7):e0197333. doi: 10.1371/journal.pone.0197333 (PMC6059400; doi:10.1371/journal.pone.0197333)
Supplement: S2 Table — (DOCX) [file pone.0197333.s016.docx]

**S2 Table. Counts of wild type (WT) and variant alleles (VA) by NGS* and variant allele frequency (VAF) for unselected ccfDNA and the size-selected fractions.**

| **ID** | **Allele** | **Unselected** | | | **Short** | | | **Medium** | | | **Long** | | |
| --- | --- | --- | --- | --- | --- | --- | --- | --- | --- | --- | --- | --- | --- |
|  |  | **WT** | **VA** | **VAF, %** | **WT** | **VA** | **VAF, %** | **WT** | **VA** | **VAF, %** | **WT** | **VA** | **VAF, %** |
| C1 | *KRAS* G13D | 6836 | 813 | 10.63 | 6625 | 1262 | 16.00 | 7585 | 1163 | 13.29 | 7323 | 581 | 7.35 |
| C2 | *BRAF* V600E | 7149 | 204 | 2.77 | 5043 | 251 | 4.74 | 7713 | 265 | 3.32 | 8058 | 158 | 1.92 |
| C3 | *KRAS* G12D | 5491 | 29 | 0.53 | 2462 | 35 | 1.40 | 4145 | 18 | 0.43 | 4759 | 13 | 0.27 |
| M1 | *BRAF* V600E | 6410 | 41 | 0.64 | 3161 | 16 | 0.50 | 5162 | 29 | 0.56 | 6363 | 31 | 0.48 |
| M2 | *BRAF* V600K | 5279 | 42 | 0.79 | 2694 | 25 | 0.92 | 4827 | 42 | 0.86 | 5448 | 38 | 0.69 |
| M3 | *BRAF* V600E | 4180 | 33 | 0.78 | 1486 | 39 | 2.56 | 2732 | 32 | 1.16 | 3657 | 16 | 0.44 |
| M4 | *BRAF* V600E | 4666 | 110 | 2.30 | 2530 | 120 | 4.53 | 3533 | 134 | 3.65 | 4772 | 63 | 1.30 |
| M5 | *BRAF* V600E | 5691 | 327 | 5.43 | 2844 | 480 | 14.44 | 4908 | 465 | 8.65 | 5010 | 126 | 2.45 |
| M6 | *BRAF* V600K | 3709 | 25 | 0.67 | 1444 | 10 | 0.69 | 3732 | 34 | 0.90 | 3983 | 30 | 0.75 |
| M7 | *BRAF* V600E | 6777 | 377 | 5.27 | 2131 | 309 | 12.66 | 5428 | 440 | 7.50 | 6654 | 269 | 3.89 |
| M8 | *BRAF* V600K | 4155 | 31 | 0.74 | 1745 | 36 | 2.02 | 2989 | 26 | 0.86 | 4710 | 25 | 0.53 |
| P1 | *KRAS* G12D | 6921 | 33 | 0.47 | 4261 | 27 | 0.63 | 7066 | 47 | 0.66 | 7526 | 23 | 0.30 |
| P2 | *KRAS* G12V | 5264 | 22 | 0.42 | 2445 | 25 | 1.01 | 4033 | 46 | 1.13 | 5385 | 18 | 0.33 |

*Unique consensus reads for family size ≥1
