# Supplementary material for: Automated size selection for short cell-free DNA fragments enriches for circulating tumor DNA and improves error correction during next generation sequencing
Source: PLoS One. 2018 Jul 25;13(7):e0197333. doi: 10.1371/journal.pone.0197333 (PMC6059400; doi:10.1371/journal.pone.0197333)
Supplement: S3 Table — (DOCX) [file pone.0197333.s017.docx]

**S3 Table. 128 genes included in next generation capture panel.**

| *ABCB1* | *ABCC9* | *ABL1* | *ADAM29* | *AFM* | *AIFM3* | *AKT1* |
| --- | --- | --- | --- | --- | --- | --- |
| *ALK* | *ANKRD36* | *APC* | *ATM* | *ATRX* | *BRAF* | *CALCR* |
| *CARD6* | *CDH1* | *CDH18* | *CDH9* | *CDHR3* | *CDKN2A* | *CDX4* |
| *CIC* | *COL1A2* | *CTNNB1* | *CXorf22* | *DCAF12L2* | *DDR2* | *DRD5* |
| *DYNC1I1* | *EDIL3* | *EGFR* | *ERBB2* | *ERBB4* | *ERCC1* | *FBXW7* |
| *FGA* | *FGFR1* | *FGFR2* | *FGFR3* | *FHL2* | *FIP1L1* | *FLT3* |
| *FOXR2* | *FRMD7* | *FUBP1* | *FZD7* | *GABRA1* | *GABRA6* | *GABRB2* |
| *GCSAML* | *GNA11* | *GNAQ* | *GNAS* | *GOLGA5* | *GPX5* | *H3F3AP4* |
| *HIST1H3B* | *HRAS* | *IDH1* | *IDH2* | *IL18RAP* | *IL1R2* | *JAK2* |
| *JAK3* | *KCNC2* | *KDR* | *KEL* | *KIT* | *KLF4* | *KRAS* |
| *KRTAP20* | *LCE4A* | *LRCC55* | *LUM* | *LZTR1* | *MAP2K1* | *MET* |
| *MMP13* | *MROH2B* | *MSH6* | *MTOR* | *NF1* | *NF2* | *NLRP5* |
| *NOTCH1* | *NOVA1* | *NRAS* | *ODF4* | *PARD6B* | *PDGFRA* | *PIK3CA* |
| *PIK3R1* | *PLCH2* | *PODNL2* | *PTEN* | *QKI* | *RB1* | *RET* |
| *RFX6* | *RPL5* | *SCN9A* | *SEMA3C* | *SEMA3E* | *SIGLEC8* | *SLC26A3* |
| *SMAD4* | *SMG5* | *SMO* | *SPO11* | *SPTA1* | *STAG2* | *STK11* |
| *SULT1B1* | *SYT14* | *TCHH* | *TERT* | *TMEM147* | *TP53* | *TPTE2* |
| *TRAF7* | *TRIM51* | *TRIM51BP* | *TRPV6* | *UGT2A3* | *VHL* | *WNT2* |
| *ZNF844* | *ZNF99* |  |  |  |  |  |
