# Supplementary material for: Automated size selection for short cell-free DNA fragments enriches for circulating tumor DNA and improves error correction during next generation sequencing
Source: PLoS One. 2018 Jul 25;13(7):e0197333. doi: 10.1371/journal.pone.0197333 (PMC6059400; doi:10.1371/journal.pone.0197333)
Supplement: S4 Table — (DOCX) [file pone.0197333.s018.docx]

**S4 Table. Eleven base-pair strings used to differentiate wild type alleles from variant alleles at allele specific locations.**

|  | **Wild Type Allele** | **Variant Allele** |
| --- | --- | --- |
| ***BRAF* V600E** | 5’-atttcactgta-3’ | 5’-atttc**T**ctgta-3’ |
| ***BRAF* V600K** | 5’-atttcactgta-3’ | 5’-atttc**TT**tgta-3’ |
| ***KRAS* G12D** | 5’-cgccaccagct-3’ | 5’-cgcca**T**cagct-3’ |
| ***KRAS* G12V** | 5’-cgccaccagct-3’ | 5’-cgcca**A**cagct-3’ |
| ***KRAS* G13D** | 5’-ctacgccacca-3’ | 5’-ctacg**T**cacca-3’ |
