# Supplementary material for: Automated size selection for short cell-free DNA fragments enriches for circulating tumor DNA and improves error correction during next generation sequencing
Source: PLoS One. 2018 Jul 25;13(7):e0197333. doi: 10.1371/journal.pone.0197333 (PMC6059400; doi:10.1371/journal.pone.0197333)
Supplement: S5 Table — (DOCX) [file pone.0197333.s019.docx]

**S5 Table. ddPCR primers and probes.**

| **Primer sequences** | | |
| --- | --- | --- |
| **Assay** | **Forward (5’>3’)** | **Reverse (5’>3’)** |
| EGFR T790M | CCTCACCTCCACCGTGCA | AGGCAGCCGAAGGGCA |
| BRAF V600E | TTTCTTCATGAAGACCTCACAGTAA | GATGGGACCCACTCCATC |
| BRAF V600K | TTTCTTCATGAAGACCTCACAGTAA | GATGGGACCCACTCCATC |
| KRAS G12D | ATGACTGAATATAAACTTGTGGTAG | GTATCGTCAAGGCACTCTT |
| KRAS G12V | ATGACTGAATATAAACTTGTGGTAG | GTATCGTCAAGGCACTCTT |
| KRAS G13D | ATGACTGAATATAAACTTGTGGTAG | GTATCGTCAAGGCACTCTT |
| **Probe sequences** | | |
| **Assay** | **Wildtype** | **Mutant** |
| EGFR T790M | /TET/T+CATC+A+C+GC/ZEN/A+GCTC/IABkFQ/ | /FAM/T+CATC+A+T+GC/ZEN/A+GC+TC/IABkFQ/ |
| BRAF V600E | /TET/AGAT+TT+C+A+C/ZEN/T+G+TAGC/IABkFQ/ | /FAM/AGAT+TT+C+T+C/ZEN/T+G+TAGC/IABkFQ/ |
| BRAF V600K | /TET/AGAT+TT+C+A+C/ZEN/T+G+TAGC/IABkFQ/ | /FAM/AGA+T+TT+C+T+T/ZEN/T+G+T+AGC/IABkFQ |
| KRAS G12D | /TET/CGCC+A+C+CAG/ZEN/CTC/IABkFQ/ | /FAM/CGCC+A+T+CA+G+CT/IABkFQ/ |
| KRAS G12V | /TET/CGCC+A+C+CAG/ZEN/CTC/IABkFQ/ | /FAM/CG+CC+A+ACA+G+CT/IABkFQ/ |
| KRAS G13D | /VIC/TTGGAGCTGGTGGCGT/NFQ/ | /FAM/CTGGTGACGTAGGCA/NFQ/ |
