# Supplementary material for: Automated size selection for short cell-free DNA fragments enriches for circulating tumor DNA and improves error correction during next generation sequencing
Source: PLoS One. 2018 Jul 25;13(7):e0197333. doi: 10.1371/journal.pone.0197333 (PMC6059400; doi:10.1371/journal.pone.0197333)
Supplement: S6 Table — Data provided summarize all ddPCR experiments performed as part of this study. (DOCX) [file pone.0197333.s020.docx]

**S6 Table. Additional essential ddPCR metrics as required according to the digital MIQE guidelines (Ref. 34). Data provided summarize all ddPCR experiments performed as part of this study.**

| **Assay** | ***EGFR* T790M** | ***BRAF* V600E** | ***BRAF* V600K** | ***KRAS* G12D** | ***KRAS* G12V** | ***KRAS* G13D** |
| --- | --- | --- | --- | --- | --- | --- |
| Cycling conditions | 10 min 95°C  15 sec 95°C 15 sec 58°C  45 sec 60°C  10 min 98°C  (50 cycles) | 10 min 95°C  15 sec 95°C 15 sec 53°C  45 sec 60°C  10 min 98°C  (45 cycles) | 10 min 95°C  15 sec 95°C 15 sec 53°C  45 sec 60°C  10 min 98°C  (45 cycles) | 10 min 95°C  15 sec 95°C  60 sec 60°C  10 min 98°C  (45 cycles) | 10 min 95°C  15 sec 95°C  60 sec 60°C  10 min 98°C  (45 cycles) | 10 min 95°C  15 sec 95°C  60 sec 60°C  10 min 98°C  (45 cycles) |
| Total PCR reaction volume | 25 μL | 25 μL | 25 μL | 25 μL | 25 μL | 25 μL |
| Individual partition volume | 5 pL | 5 pL | 5 pL | 5 pL | 5 pL | 5 pL |
| Total intact partitions analyzed per reaction; Mean ± SD (Range) | 4216327  ± 209387  (3603453 -  4966439) | 3961420  ± 235988  (3214109 -  4397148) | 3786233  ± 428023  (2326784 -4342012) | 4093765  ± 221102  (3580018 -  4572790) | 4023374  ± 190681  (3497570 -  4341072) | 2572434  ± 774945  (1270067 -  3905166) |
| Effect reaction volume analyzed (μL); Mean ± SD (Range) | 21.1  ± 1.05  (18.0 - 24.8) | 19.8  ± 1.18  (16.1 - 22.0) | 18.9  ± 2.15  (11.6 - 21.7) | 20.5  ± 1.11  (17.9 -  22.9) | 20.1  ± 0.95  (17.5 -  21.7) | 12.9  ± 3.87  (6.4 -  19.5) |
| Mean target copies per partition; Mean ± SD (Range) | 0.0013  ± 0.00065  (0.0003 -  0.0053) | 0.0011  ± 0.00036  (0.0002 -  0.0028) | 0.0011  ± 0.00058  (0.0005 -0.0032) | 0.0011 ±  0.00058  (0.0004 - 0.0039) | 0.0011  ± 0.00046  (0.0003 -  0.0027) | 0.0014  ± 0.00067  (0.0003 -  0.0030) |
